# A CTP Synthase Undergoing Stage-Specific Spatial Expression Is Essential for the Survival of the Intracellular Parasite *Toxoplasma gondii*

**DOI:** 10.3389/fcimb.2018.00083

**Published:** 2018-03-22

**Authors:** Heidy Y. Narvaez-Ortiz, Andrea J. Lopez, Nishith Gupta, Barbara H. Zimmermann

**Affiliations:** ^1^Departamento de Ciencias Biologicas, Universidad de los Andes, Bogota, Colombia; ^2^Department of Molecular Parasitology, Faculty of Life Sciences, Humboldt University, Berlin, Germany

**Keywords:** *Toxoplasma gondii*, CTP synthase, pyrimidine, nucleotide biosynthesis, *cytoophidium*

## Abstract

Cytidine triphosphate synthase catalyzes the synthesis of cytidine 5′-triphosphate (CTP) from uridine 5′-triphosphate (UTP), the final step in the production of cytidine nucleotides. CTP synthases also form filamentous structures of different morphologies known as *cytoophidia*, whose functions in most organisms are unknown. Here, we identified and characterized a novel CTP synthase (*Tg*CTPS) from *Toxoplasma gondii*. We show that *Tg*CTPS is capable of substituting for its counterparts in the otherwise lethal double mutant (*ura7*Δ *ura8*Δ) of *Saccharomyces cerevisiae*. Equally, recombinant *Tg*CTPS purified from *Escherichia coli* encodes for a functional protein in enzyme assays. The epitope-tagged *Tg*CTPS under the control of its endogenous promoter displays a punctate cytosolic distribution, which undergoes spatial reorganization to form foci or filament-like structures when the parasite switches from a nutrient-replete (intracellular) to a nutrient-scarce (extracellular) condition. An analogous phenotype is observed upon nutrient stress or after treatment with a glutamine analog, 6-diazo-5-oxo-L-norleucine (DON). The exposure of parasites to DON disrupts the lytic cycle, and the *Tg*CTPS is refractory to a genetic deletion, suggesting an essential requirement of this enzyme for *T. gondii*. Not least, this study, together with previous studies, supports that CTP synthase can serve as a potent drug target, because the parasite, unlike human host cells, cannot compensate for the lack of CTP synthase activity.

## Introduction

*Toxoplasma gondii*, the causative agent of Toxoplasmosis, affects approximately one third of the world's population. It is usually asymptomatic in healthy individuals, but can lead to severe problems in the fetus and in immunocompromised or immunosuppressed patients, such as those infected with HIV (Montoya and Liesenfeld, 2004). The current treatments against toxoplasmosis display side effects and adverse reactions in many patients (Haverkos, 1987; Leport et al., 1988). In addition, studies show that the effectiveness of prenatal treatment is uncertain (Gilbert, 2001). Thus, it is important to seek new targets for drug design.

The *de novo* pyrimidine biosynthetic pathway in *T. gondii* is a potential target for new drugs, because it is required for the parasite's virulence and survival (Fox and Bzik, 2002; Hyde, 2007). The rate limiting step in this pathway is catalyzed by CTP synthase, which catalyzes the formation of CTP, an essential biomolecule (Long et al., 1970; Endrizzi et al., 2005). CTP serves as a building block for nucleotides and nucleic acids (Hatse et al., 1999) and is required for protein glycosylation (Denecke and Kranz, 2009). Moreover, it acts as a high energy molecule during lipid biogenesis (Liu et al., 2004; Chang and Carman, 2008) and participates in cellular communication processes (Sellmeier et al., 2013). Indeed, it is a recognized potential target for drug development to treat diseases such as cancer (Williams et al., 1978; Verschuur et al., 1998; Hansel et al., 2003) and infections caused by viruses (De Clercq, 2009), bacteria (Wylie et al., 1996a,b; Mori et al., 2015), and parasites (Lim et al., 1996; Hendriks et al., 1998; Hofer et al., 2001).

Recently, it has been shown that CTP synthase can form filaments in a wide range of organisms, from prokaryotes to eukaryotes, for example *Caulobacter crescentus* (Ingerson-Mahar et al., 2010), *Escherichia coli* (Barry et al., 2014), *Schizosaccharomyces pombe* (Ingerson-Mahar et al., 2010; Zhang et al., 2014), *Saccharomyces cerevisiae, Drosophila melanogaster, D. virilis, D. pseudoobscura* (Liu, 2010), *Rattus norvegicus* (Noree et al., 2010) and mammalian cells (Carcamo et al., 2011). In *C. crescentus*, the filaments play a cytoskeleton-like role in maintaining the bacterium's curved morphology, nevertheless, in most organisms, their function remains unknown. The dynamics of the formation of CTP synthase structures, designated as *cytoophidia* (Greek *cyto* = cell and *ophidia* = serpents), rods and rings (RR) structures, or CTPS structures, seem to be species-specific. The process is regulated by nutrient deficit, intracellular levels of nucleotides, progress of the cell cycle, and the presence of inhibitors.

In mammalian cells, mainly in cancer cell lines, some RR structures also contain inosine monophosphate dehydrogenase 2 (IMPDH2) as a major component (Carcamo et al., 2011). They are strongly induced by treatment with either CTP synthase inhibitors such as 6-diazo-5-oxo-L-norleucine (DON), acivicin and 3′-deazauridine, or IMPDH2 inhibitors such as ribavirin and mycophenolic acid (Ji et al., 2006; Carcamo et al., 2011; Chen et al., 2011; Chang et al., 2015). Interestingly, most primary cell lines present RR structures only under treatment with CTP synthase or IMPDH2 inhibitors (Carcamo et al., 2011). The RR structures are not associated with any other known subcellular structure (Thomas et al., 2012), although they are present in the nucleus as well as in the cytosol (Carcamo et al., 2014).

This study identified and characterized the CTP synthase from *T. gondii* (*Tg*CTPS). Unlike mammals, *T. gondii* and other parasites have a limited capacity to salvage pyrimidines, most relevantly cytidine (Hofer et al., 2001; Yuan et al., 2005; Garavito et al., 2015), therefore *Tg*CTPS represents an excellent anti-parasitic target.

## Results

### *Tg*CTPS encodes an active CTP synthase

**Figure 1:**
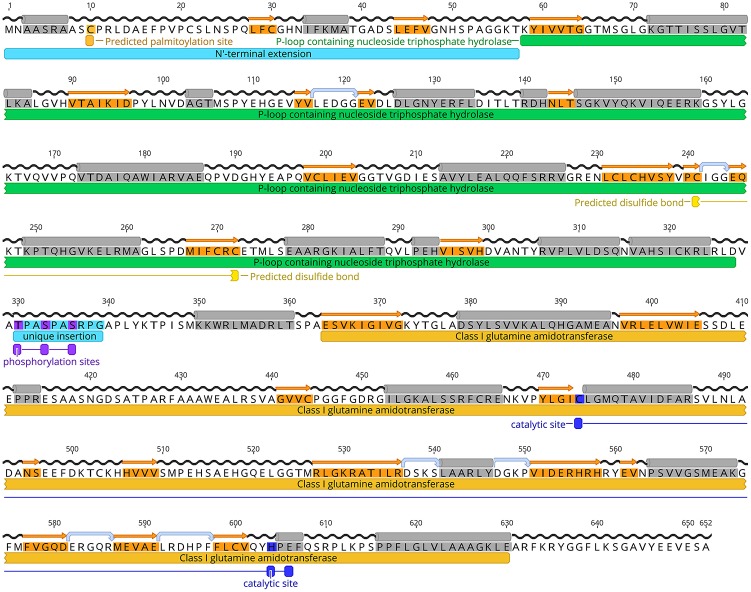
*Tg*CTPS amino acid sequence. A consensus of secondary structure predictions is shown above the sequence. α- helices are highlighted in gray, strands in orange, hairpins in light blue and coils in black. The conserved domains detected in the *Tg*CTPS sequence are indicated by bars below the sequence. They include a P-loop containing nucleoside triphosphate hydrolase domain at the N-terminus (green) and a class I glutamine amidotransferase domain at the C-terminus (yellow). A palmitoylation site is predicted in the N-terminal extension. Cysteines 241 and 272, which form a disulphide bond, are depicted in light yellow. According to the phosphoproteomic data from ToxoDB, three residues are phosphorylated *in vivo* in tachyzoites that are located in an insertion unique to *Tg*CTPS. The catalytic triad (Cys474-His604-Glu606) is indicated below the sequence in blue. Figure was generated using Geneiuos 9.1, available from http://www.geneious.com.

Our bioinformatic searches identified a single CTP synthase gene in *T. gondii* (TGGT1_299210) that consists of 6 exons and 5 introns, and encodes a putative protein of 652 amino acids (toxodb.org). The open reading frame was cloned, confirmed by sequencing and submitted to GenBank (JN847214.1). The predicted amino acid sequence of *Tg*CTPS contained two conserved domains, an N-terminal synthase domain (a P-loop containing nucleoside triphosphate hydrolase) and a C-terminal glutaminase domain with the conserved catalytic triad, Cys474—His604—Glu606 of a type I amidotransferase (Massière and Badet-Denisot, 1998) (Figure 1). *Tg*CTPS contained a unique N-terminal extension (57 amino acids), an insertion of 10 residues from T330 to Gly339 that is not present in CTP synthases from other organisms, and an insertion of 7 residues from A416 to S423, adjacent to a helix found in the glutaminase domain of eukaryotic CTP synthases (Lynch et al., 2017) (Supplementary Figure 1).

**Figure 2:**
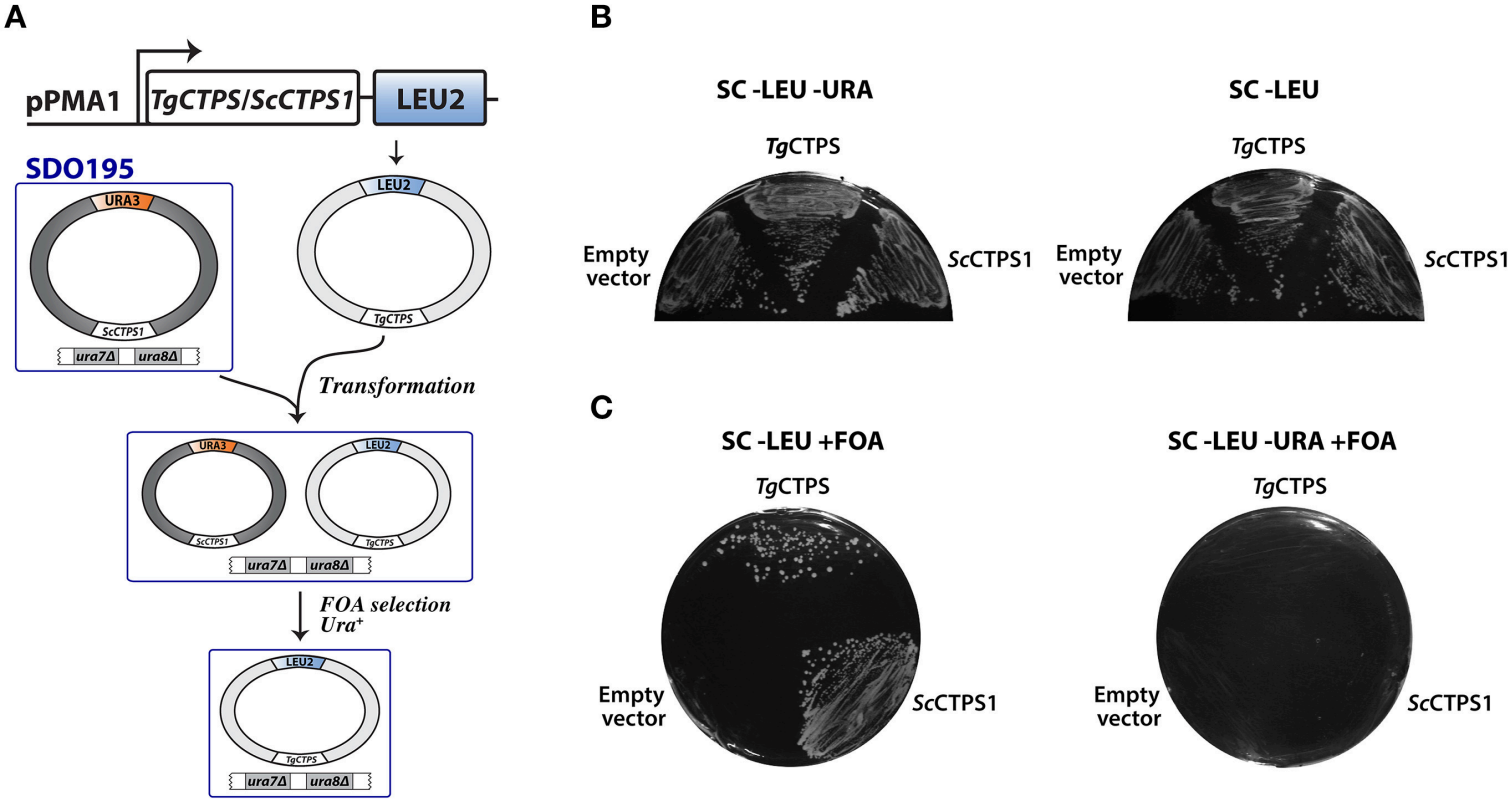
Functional complementation of yeast CTP synthase double mutant by *Tg*CTPS. **(A)** Schematic representation of the plasmid shuffling technique applied to test *Tg*CTPS function *in vivo* in *S. cerevisiae*. **(B)** The SDO195 strain was transformed with a *LEU2* plasmid having the *TgCTPS* coding sequence. Resultant transformants were plated on SC medium without leucine but containing uracil, and SC medium without uracil or leucine to select colonies carrying the two plasmids. **(C)** A colony of each transformant, carrying two plasmids, was streaked out on SC −LEU +FOA and SC −LEU −URA +FOA media. *Sc*CTPS1 was used as a positive control and the empty vector was the negative control. Results are representative of three independent experiments.

To test whether the putative *Tg*CTPS sequence encoded an active enzyme, we performed a complementation assay in *S. cerevisiae* SDO195. This yeast strain lacks the two endogenous CTP synthases, and therefore requires the presence of a plasmid expressing an active CTP synthase to grow (Figure 2) (Ozier-Kalogeropoulos et al., 1994). The strain is also deficient in LEU2 and URA3, enzymes of leucine and pyrimidine biosynthesis, which makes it auxotrophic for leucine and uracil, and consequently facilitates genetic manipulation. SDO195 harbors a plasmid (*Sc*CTPS1/URA3) expressing yeast CTPS1 and URA3 to permit its selection on uracil-free media. A plasmid shuffling approach was used to replace this plasmid with another plasmid (*Tg*CTPS/LEU2) expressing *Tg*CTPS and LEU2. Positive and negative controls were a plasmid expressing *Sc*CTPS1 (*Sc*CTPS1/LEU2), and the empty vector, respectively. First, SDO195 was transformed with *Tg*CTPS/LEU2, and cells were selected on plates containing synthetic complete (SC) medium without leucine or uracil, or on SC medium without leucine but containing uracil (Figure 2B). The colonies selected in the latter medium contained both plasmids, *Tg*CTPS/LEU2 and ScCTPS1/URA3, as confirmed by PCR (data not shown). Next, to eliminate cells expressing ScCTPS1/URA3, we employed a counter-selection strategy using 5′-fluoorotic acid (5′-FOA), which is based on the capability of URA3-expressing cells to metabolize this compound into a toxic uracil analog, leading to cell death (Figure 2C). The loss of the *Sc*CTPS1/URA3 was confirmed by PCR (data not shown) and by auxotrophy for uracil (Figure 2C). Unlike the negative control, the plasmid expressing *Tg*CTPS, as well as the positive control expressing *Sc*CTPS1, permitted growth of *S. cerevisiae* on the selective medium, which confirmed that the parasite protein was functional, and capable of substituting for the yeast protein *in vivo*.

### The *Tg*CTPS gene is essential for parasite survival

In *T. gondii, de novo* pyrimidine biosynthesis is a functional and essential pathway (Fox and Bzik, 2002; Fox et al., 2011). We used different strategies to produce a tachyzoite *TgCTPS* mutant. The first strategy consisted of the destabilization of *TgCTPS* expression by Cre-mediated displacement of its 3′-UTR (Supplementary Figure 2A). Parasites (Δ*ku80::DiCre*) (Andenmatten et al., 2013) were transfected with a construct for 3′HA tagging of the *TgCTPS* gene and selected in medium containing mycophenolic acid (MPA) and xanthine (XAN). Unfortunately, transfected parasites did not survive when the selection was applied. Transfection experiments were conducted three times without any success, which indicated that *TgCTPS* gene is regulated by its own 3′-UTR.

In a second approach, we attempted a swap of the endogenous *TgCTPS* locus for a *TgCTPS* cDNA, which was flanked by Cre-loxP sites and regulated by the *TgCTPS*−5′-UTR (1.5 kb) and *TgCTPS*-3′-UTR (0.67 kb). A weak recombination-specific amplification was observed in the pool of transfected parasites (Supplementary Figure 2B), however, in individual clones, the PCR identified only single crossover events at the 5′- or 3′-end, indicating that the endogenous gene was retained in all cases.

We therefore decided to make a conditional mutant of *TgCTPS* using a tetracycline-regulatable expression system. The proper insertion of the drug-repressible *TgCTPS-myc* ORF was tested by western blot, confirming the expression of the full-length tagged protein (Supplementary Figure 2C). The merodiploid strain (*T*g*CTPS*pTet*/TgCTPS*) grown with anhydrotetracycline (ATc) for 40 h showed a decrease in the expression of *Tg*CTPS-c-myc (Supplementary Figure 2C). We then designed the *TgCTPS* KO construct harboring CAT selection marker (Kim et al., 1993). Parasites transfected with *TgCTPS_KO_CAT* survived chloramphenicol selection (Supplementary Figure 2C). Recombination-specific PCR to assess the genomic integration showed that some of the isolated clones were positive for 5' crossover whereas others for 3' crossover, but a mutant undergoing double crossover was never observed (data not shown). Transfection experiments and PCR screening were carried out at least three times, without observing double crossovers. Taken together, these results indicate that CTP synthase is tightly regulated and likely essential for parasite survival. Likewise, out multiple attempts to delete the gene locus by double homologous recombination were also unsuccessful, suggesting an essential function of CTP synthase in *T. gondii*.

### Purification of active recombinant *Tg*CTPS

We expressed full-length *Tg*CTPS with a 6xHis tag at the N-terminus in *E. coli* under standard culture conditions (Supplementary Figure 3). Purification of this enzyme by cobalt-affinity chromatography led to the recovery of a very small amount (≤ 0.02 mg per 1L cell culture) of active, soluble, full-length *Tg*CTPS, however with low purity (Supplementary Figure 3B). Altering the induction and purification conditions increased the yield (Supplementary Figures 3C,D), but resulted in inactive protein (data not shown). Therefore, we isolated *Tg*CTPS inclusion bodies from cell extract by centrifugation, solubilized them in the presence of urea, and refolded by a matrix-assisted method (Tsumoto et al., 2003; Dashivets et al., 2009), which allowed us to recover active full-length refolded protein of approximately 80% purity with a yield of 0.75 mg/L of cell culture (Supplementary Figure 3E).

Dybas and coworkers performed a proteomic experiment where membrane and cytosolic fractions of extracellular tachyzoites were analyzed by mass spectrometry (Dybas et al., 2008). Interestingly, two peptides of the unique N-terminus of *Tg*CTPS were found in the membrane fraction, while 17 peptides from the rest of the *Tg*CTPS sequence were found in the cytosolic fraction (toxodb.org). In light of these data, we expressed a version of 6xHis-*Tg*CTPS lacking the first 57 residues, and obtained a significant amount of active, soluble, truncated, *Tg*CTPS, which was ten-fold more soluble than the full-length protein, at a yield of ≤ 0.2 mg/L cell culture (Supplementary Figure 3F). The removal of this peptide increased the protein's solubility in *E. coli* without affecting the enzymatic activity *in vitro* as described below.

### Kinetic characterization of *Tg*CTPS activity

**Figure 3:**
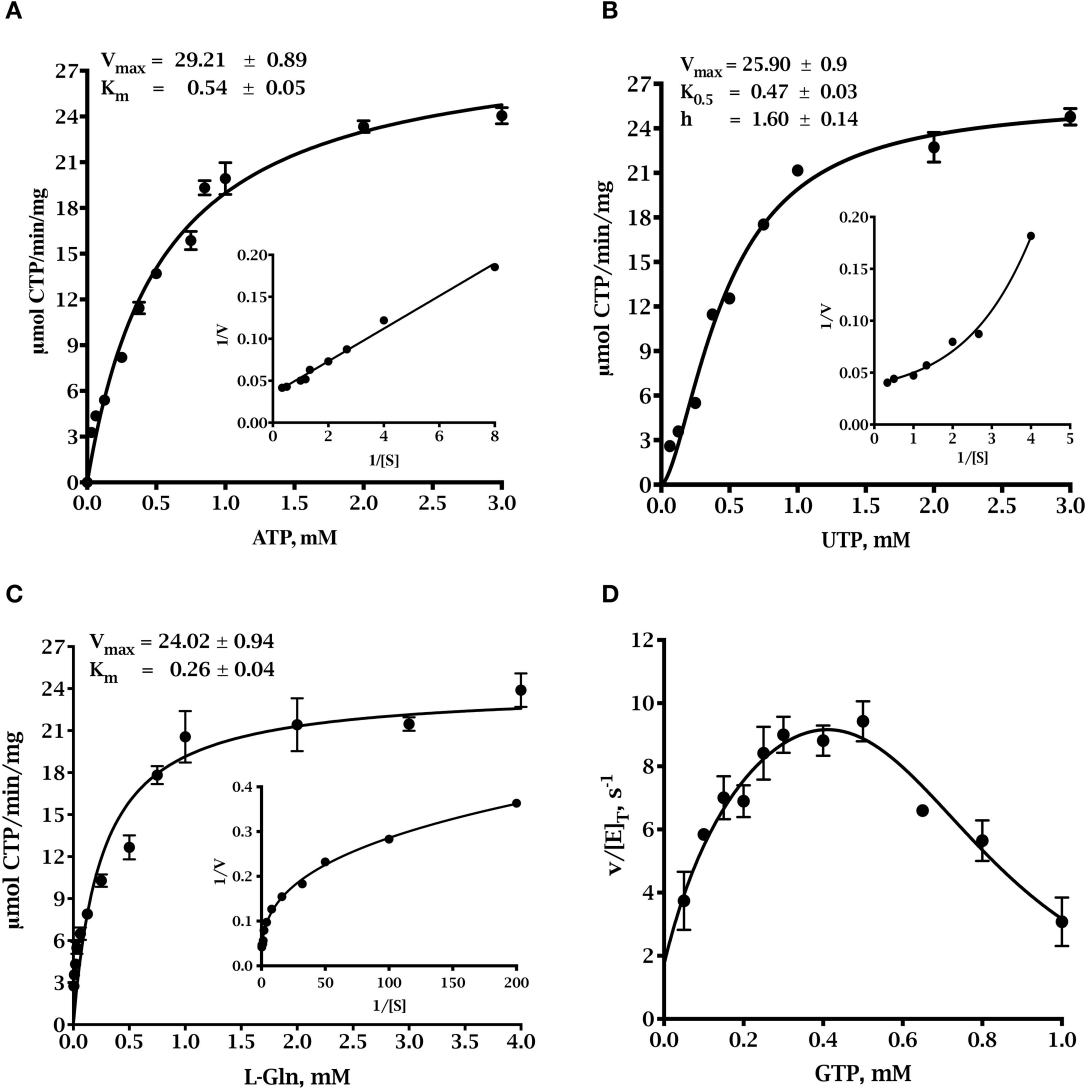
Steady state kinetics of full-length refolded *Tg*CTPS. **(A)** ATP saturation curve. Data were fitted to the Michaelis-Menten equation, ***V*=*V***_***max***_ × [***S***]**÷**(***K***_***m***_+[***S***]). **(B)** UTP saturation curve. Parameters were calculated by fitting the data to the allosteric sigmoidal equation, $Y=V_{max}\;\times \;{\left[S\right]}^{h}÷(K_{0.5}^{h}+\;{\left[S\right]}^{h})$. **(C)** Glutamine saturation curve. Data were fitted to the Michaelis-Menten equation. **(D)** The effect of GTP on the glutaminase-dependent *Tg*CTPS activity. In panels **(A-C)**, the insets show the corresponding double reciprocal plots.

The synthesis of CTP by CTP synthase requires the substrates ATP, UTP and glutamine. GTP participates in the reaction as a positive effector (Lieberman, 1956; Levitzki and Koshland, 1969). The kinetic parameters for the three *Tg*CTPS recombinant enzyme preparations are shown in Figure 3 and Table 1. The UTP saturation curves produced by all three showed similar sigmoidal behaviors (data not shown) and kinetic parameters (Table 1), supporting the idea that the refolding process did not significantly affect the activity (Table 1) as reported for other CTP synthases (Yuan et al., 2005). The full-length, refolded *Tg*CTPS was used for subsequent kinetic studies, because of its higher yield.

**Table 1:** Kinetic parameters of CTP synthases from different organisms.

**Organism**	**Substrate**	**V_max_ (U mg^−1^)**	**K_m_ (mM)**	**K_0.5_ (mM)**	**k_cat_ (s^−1^)**	**k_cat_ / K_m_ (s^−1^ mM^−1^)**	**Reference**
*T. gondii* (full length refolded)^a^	L-Gln	24.0 ± 0.9	0.26 ± 0.04		29.6	114	This work
	UTP	25.9 ± 0.9		0.47 ± 0.03 *h* = 1.60 ± 0.14	31.9	–	
	ATP	29.2 ± 0.9	0.54 ± 0.05		36	66.7	
*T. gondii* (truncated) ^a^	UTP	31.3 ± 2.0		0.41 ± 0.06 *h* = 1.14 ± 0.15	38.6	104.3	This work
*T. gondii* (full length) ^a^	UTP	24.9 ± 3.0		0.41 ± 0.09 *h* = 1.40 ± 0.37	30.7	105.9	This work
*E. coli*	L-Gln	–	0.35 ± 0.06	–	6.1	17.8 ± 2.3	Lunn and Bearne, 2004; MacLeod et al., 2006
	UTP	–	−	0.28 ± 0.04 *h* = 1.26 ± 0.14	13.7	48.8	
	ATP	–	−	0.49 ± 0.02 *h* = 2.20 ± 0.12	12.8	25.9	
*S. cerevisiae (URA7)*	UTP	0.37		0.11 *h* = 1.4	–	–	Park et al., 2003
	ATP	0.28		0.45	–	–	
*Homo sapiens* (CTP 1)	L-Gln	–	0.027 ± 0.009	–	–	–	Chang et al., 2007; Kassel et al., 2010
	UTP	–	0.59 ± 0.24	–	≈ 0.12	–	
	ATP	–	0.17 ± 0.11	–	-	–	
*Homo sapiens* (CTP 2)	L-Gln	–	0.1 ± 0.04	–	-	–	
	UTP	–	0.19 ± 0.08	–	–	–	
	ATP	–	0.06 ± 0.02	–	–	–	

*The values of TgCTPS kinetic constants were determined in triplicate and the averages are reported. The reported errors are standard deviations. One unit is defined as the conversion of 1 μmol of substrate per minute at 37°C*.

a *The values were calculated with GraphPad Prism version 6.0e according to the best-fitted model*.

The ATP saturation curve was fit to the Michaelis-Menten equation (Figure 3A). However, the UTP saturation curve exhibited sigmoidal behavior suggesting positive cooperativity, which was confirmed by the shape of the curve in the double reciprocal plot and by the Hill coefficient value > 1 (Figure 3B). Positive cooperativity for UTP has been shown for other CTP synthases, such as enzymes from *E. coli, S. cerevisiae, Lactococcus lactis*, etc. (Long and Pardee, 1967; Yang et al., 1994; Nadkarni et al., 1995; Wadskov-Hansen et al., 2001). The L-glutamine saturation curve appeared to be hyperbolic, but the curve of the double reciprocal plot was concave downward instead of linear (Figure 3C), and the Hill coefficient was less than 1, which indicated negative cooperativity in the binding of L-glutamine to the active site (Levitzki and Koshland, 1972; Segel, 1993). Negative cooperativity for L-glutamine has also been described for *Ec*CTPS (Levitzki and Koshland, 1972) and *Sc*CTP1S (Yang et al., 1994). The activity of *Tg*CTPS depended on GTP concentration, as observed for other CTP synthases (Levitzki and Koshland, 1972; MacDonnell et al., 2004; Endrizzi et al., 2005; Steeves and Bearne, 2011). We observed activation at GTP <0.5 mM, and inhibition at >0.5 mM (Figure 3D). The turnover number of *Tg*CTPS was approximately three-fold higher than that of *Ec*CTP (Lunn and Bearne, 2004) and approximately 160-fold higher than that of the *Hs*CTPS (Chang et al., 2007; Kassel et al., 2010). The catalytic efficiency, *k*_cat_/*K*_m_, of *Tg*CTPS was also higher than those observed for *Ec*CTPS and *Hs*CTPS (Table 1) (Lunn and Bearne, 2004; Chang et al., 2007; Kassel et al., 2010). The physiological concentrations of ATP in tachyzoites range from ≈0.8 to 1.1 mM (Pace et al., 2011), suggesting a maximal rate of ≈70% for *Tg*CTPS *in vivo*. Our kinetic characterization of *Tg*CTPS constitutes the first step toward understanding the regulation of cytidine pools in *T. gondii*.

We tested the effect of a glutamine analog DON, a known inhibitor of CTP synthases, on *Tg*CTPS full-length refolded protein, and found an IC50 of ≈ 0.04 μM (Supplementary Figure 4). A concentration of 1 μM DON completely eliminated the measurable enzyme activity (Supplementary Figure 4).

### *Tg*CTPS forms foci-like structures depending on its expression levels and the parasitic stage

**Figure 4:**
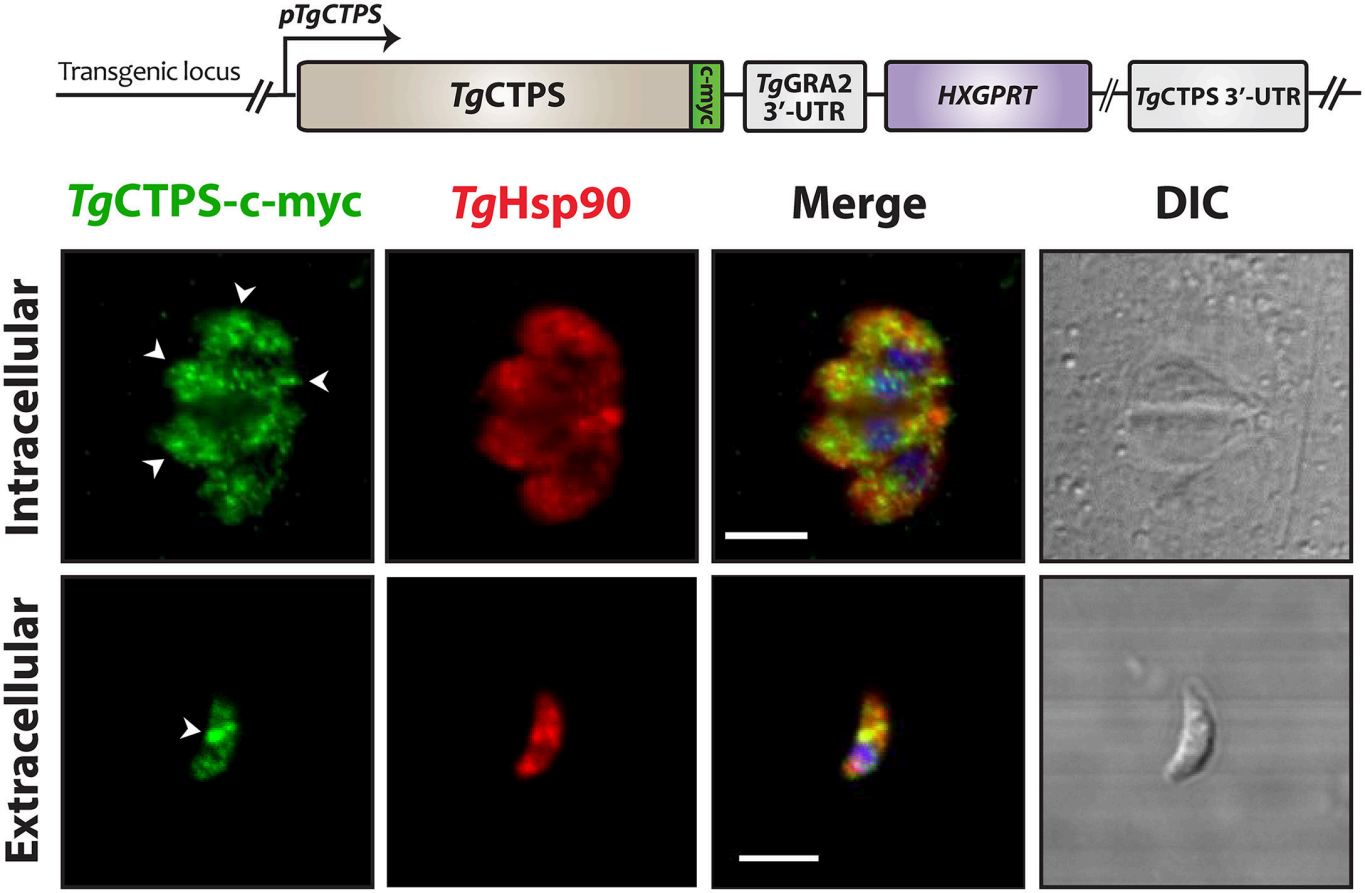
The subcellular localization of *Tg*CTPS in tachyzoites. *Tg*CTPS expressed at endogenous levels. Immunostaining was performed using mouse anti-c-myc (green) and rabbit anti-TgHsp90 (red) antibodies. *Intracellular:* HFF cells were infected with transfected parasites and fixed 27 hpi. *Extracellular:* Fresh extracellular parasites were fixed. The *Tg*CTPS punctate distribution is indicated by arrowheads (white). DIC: differential interference contrast. Scale bars: 5 μm.

To determine whether *Tg*CTPS was able to form filament-like structures in *T. gondii* tachyzoites, we generated an epitope-tagged construct, and stable transgenic parasites expressing it were selected for localization studies (Figure 4). This construct allowed expression of a myc-tagged version of *Tg*CTPS under control of its own promoter region (*pTgCTPS*). The resulting strain, *RH TgCTPS_c-myc-HX*, expressed the protein at endogenous levels (Figure 4). Immunofluorescence experiments showed *Tg*CTPS formed foci-like structures in both intracellular and extracellular parasites, although this pattern was more pronounced in the latter (Figure 4). These results suggest that the ability to form structures is conserved in *Tg*CTPS, and the frequency varies according to the infectious stage of the parasite. We classified the *Tg*CTPS structures observed as follows: *foci* are approximately circular structures, *small foci* are <0.5 μm in diameter and *large foci* are >0.5 μm in diameter; *filament-like structures* are elongated structures, with 0.8 to 3 μm length, and 0.2–0.5 μm width.

### The glutamine analog DON alters localization of *Tg*CTPS

**Figure 5:**
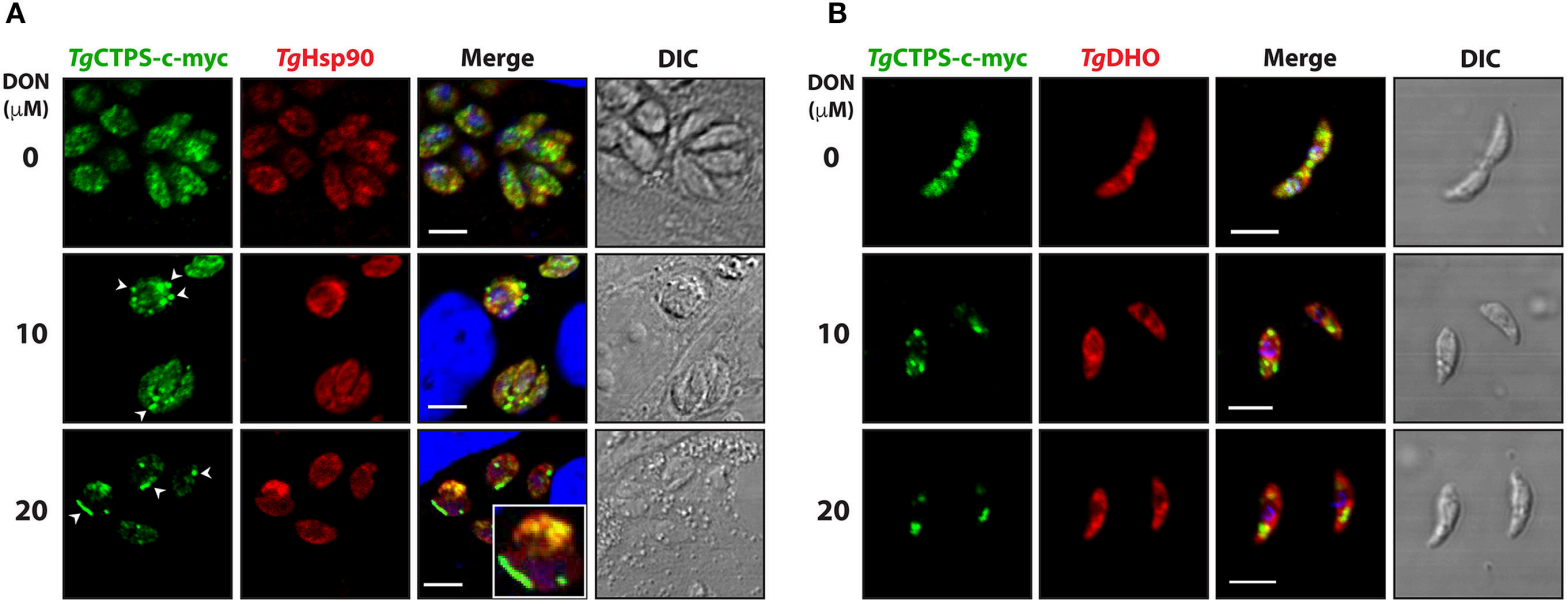
DON concentration-dependent localization of *Tg*CTPS in intracellular parasites **(A)** and extracellular parasites **(B)**. **(A)** Immunofluorescence experiments were performed with infected HFF cells cultured in the presence of variable concentrations of DON for 27 h. *Tg*CTPS structures, such as larger foci and filament-like structures, are indicated by arrowheads (white). Inset shows a zoom-in (3x magnified) for a *Tg*CTPS filament-like structure **(B)** Immunofluorescence experiments were performed with fresh extracellular parasites incubated for 6 h in variable concentrations of DON. Scale bars: 5 μm.

**Figure 6:**
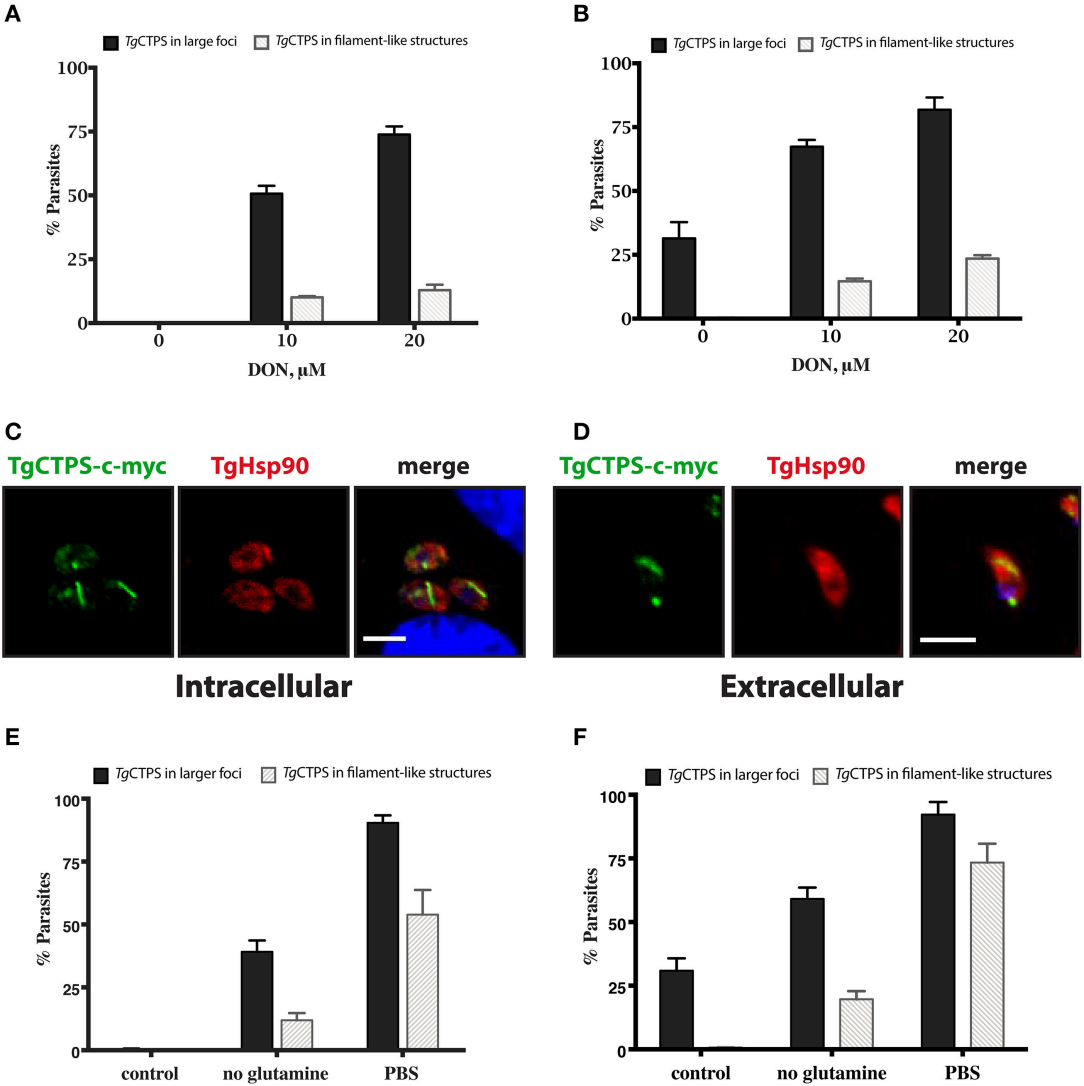
The formation of *Tg*CTPS structures in the presence of DON or in nutrient depletion**. (A,B)** The percentage of parasites showing *Tg*CTPS structures was calculated based on the total number of cells for each condition. **(A)** Intracellular parasites were allowed to invade and replicate in medium containing DON for 27 h, then IFAs were performed and *Tg*CTPS structures were counted. The bars show the mean ± SD for three independent assays, each with at least 100 vacuoles **(B)** Extracellular parasites were incubated in medium containing DON for 4–6 h. Three independent assays, each with at least 100 parasites, were evaluated; mean ± SD. **(C,D)** Distribution of *Tg*CTPS in the presence of DON. **(C)** Intracellular parasites grown in the presence of 20 μM DON for 27 h. **(D)** Extracellular parasites incubated in the presence of 20 μM DON for 3 h. **(E,F)** The percentage of parasites showing *Tg*CTPS structures was calculated based on the total number of cells for each condition. **(E)** Intracellular parasites were allowed to invade and replicate in standard media (control), DMEM media without glutamine or in PBS, then IFAs were performed and *Tg*CTPS structures were counted. **(F)** Extracellular parasites were incubated in standard media (control), DMEM media without glutamine or in PBS for 4 h.

To examine whether DON affects the dynamics of *Tg*CTPS localization, the *RH TgCTPS_c-myc-HX* strain was grown in the presence of variable concentrations of DON ranging from 2 μM to 20 μM. Changes in *Tg*CTPS localization were evaluated by IFA in intracellular (Figure 5A) and extracellular parasites (Figure 5B). DON promoted the formation of *Tg*CTPS structures that began as simple foci in the cytosol (in absence or at low concentrations of DON) and then assembled into large foci, donuts or filament-like structures at high concentrations of the drug (Figure 5). Quantification of this effect revealed that approximately 30% of the extracellular parasites that were cultured in the absence of DON exhibited large foci-like structures, while intracellular parasites did not exhibit these structures (Figures 6A,B). When parasites were treated with 20 μM DON more than 23% of the extracellular, and approximately 13% of the intracellular parasites, exhibited large filament-like structures, whose lengths ranged from >1 to 3 μm (Figures 6C,D). Tachyzoites are approximately 6 μm in length and 2 μm in diameter (Dubey et al., 1998), meaning that the *Tg*CTPS filament-like structures occupied up to half the length of the parasite. In most cases, there was only one *Tg*CTPS filament-like structure per cell, usually oriented to the apical end. In addition, parasites harboring a filament-like structure also presented a small punctum, apparently localized in the nucleus or between the nucleus and the posterior end.

We concluded that DON stimulated *Tg*CTPS assembly in a concentration-dependent manner causing an increase in the frequency of filament-like structure formation, as observed for other eukaryotic CTP synthases (Carcamo et al., 2011; Chen et al., 2011; Calise et al., 2014). The *Tg*CTPS polymerization appears to be promoted not only subsequent to the release of tachyzoites from the host cell, but also upon inhibition of the CTP synthase by DON.

### DON impairs the lytic cycle of *T. gondii*

**Figure 7:**
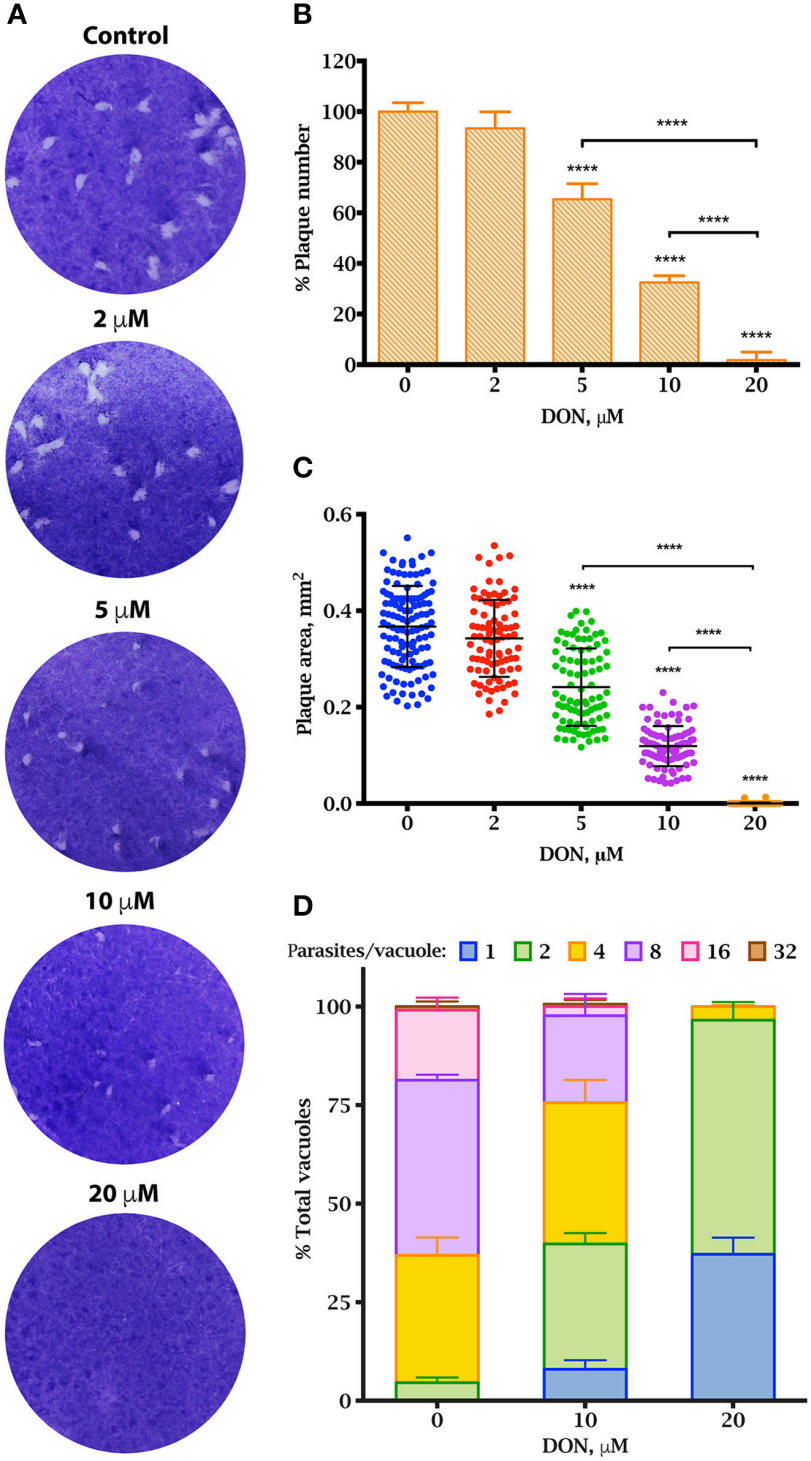
Effect of DON in parasite propagation in culture. *Plaque assay showing a reduction in T. gondii proliferation in vitro in the presence of DON* **(A–C)**. **(A)** Parasites were grown on human foreskin fibroblasts in the presence or variable concentrations of DON for 7 days before fixation and staining with crystal violet. Representatives of three independent experiments for each concentration are shown. **(B)** The number of plaques observed in the experiment from panel **A**. The percentage was calculated based on total number of plaques observed in the control. **(C)** Sizes of plaques in mm^2^. *Replication assay under DON treatment* **(D)** The rate of intracellular growth was monitored 27 h after infection by counting the number of parasites per vacuole. Significant differences were estimated by one-way ANOVA and Dunnett's test, ^****^P < 0.0001; mean ± SD (*n* = 3).

The lytic cycle of *T. gondii* underlies the establishment of the acute infection *in vivo* (Blader et al., 2015). To investigate whether DON exhibited an anti-parasitic activity, we performed plaque assays, which recapitulate the successive rounds of lytic cycles *in vitro*. Confluent monolayers of HFF cells were infected with *RH TgCTPS_c-myc-HX* strain and grown in medium containing variable concentrations of DON for 7 days, and then stained with crystal violet. Plaques were identified as clear zones indicating host cell lysis on a background of dye-stained host monolayer (Figure 7A). We observed that the number and size of plaques were significantly reduced in cultures treated with concentrations ≥5 μM of DON (Figures 7B,C), which appeared to cause a strong endodyogeny defect that prevented daughter cells to be completely separated during cell division (Figure 7D). This was also evident in the replication rates of the parasites cultured in the presence of DON, where vacuoles contained only one, two or four parasites, while vacuoles in control samples contained up to 32 parasites (Figure 7D). Similar results were obtained when experiments were carried out with tachyzoites of the parental strain of *T. gondii* (data not shown). It is important to note that approximately the same percentage of cells were infected in absence or presence of DON, thus the inhibitor apparently did not affect invasion.

DON treatment of cultures resulted in morphologically altered parasites, referred to as “aberrant” parasites herein. The loss of the typical rosette organization was observed in intracellular parasites and the parasitophorous vacuole of the aberrant parasites were amorphous with a large vacuolar space (Figure 5A). In both intracellular and extracellular parasites, aberrant morphology was remarkably prevalent (Supplementary Figure 6A). Extracellular parasites were blunted and spherical compared to the control (Supplementary Figure 6B). DON is not specific for CTP synthases, and can inhibit other glutamine amidotransferases (Kisner et al., 1980) (Supplementary Figure 7A). To test whether growth impairment was due to DON inhibition of carbamoyl phosphate synthase II, or GTP synthase, we compared the replication rates of DON-treated cultures in the presence or absence of uracil or guanine, respectively. Supplementation did not significantly mitigate the effects of DON on the lytic cycle, suggesting that *Tg*CTPS is the primary target of the drug (Supplementary Figure 7C).

## Discussion

Nucleotide metabolism is energetically costly and usually tightly controlled (Berg et al., 2002). CTP synthase is regulated at the transcriptional level (Wylie et al., 1996a; Meng and Switzer, 2001; Jørgensen et al., 2003), allosterically at the enzymatic level (Willemoes and Larsen, 2003; MacDonnell et al., 2004; Endrizzi et al., 2005; Willemoes et al., 2005), and by post-translational modifications (Park et al., 2003; Chang et al., 2007; Choi and Carman, 2007; Kassel et al., 2010). The assembly of CTP synthase into *cytoophidia* has been proposed to be an additional mechanism of regulation, in which the enzymatic activity is controlled by storing the protein in polymers composed of inactive or less active protein in *E. coli, S. cerevisiae* and *Drosophila* (Aughey et al., 2014; Barry et al., 2014; Noree et al., 2014). Nevertheless, filaments containing active CTP synthase have also been proposed for *Drosophila* (Strochlic et al., 2014). Recently, Lynch et al. (2017) used cryoelectron microscopy and X-ray crystallography to show that the conformation of *E. coli* CTP synthase in filaments have reduced affinity for substrates. In contrast, they found a different architecture for filaments composed of human CTP synthase 1, where the protein was present in an active conformation (Lynch et al., 2017). Although *Tg*CTPS is highly expressed in extracellular as well as in intracellular tachyzoites, our data show that filament-like structure occur in a stage-dependent manner in *T. gondii*. We hypothesize that when the parasite is outside the host cell, it requires a low CTPS activity, likely regulated by protein assembly into filaments acting as the transient enzyme storage. In contrast, intracellular parasites replicating within vacuoles should require a congruently high CTPS activity to satisfy the nucleotide and lipid biogenesis. This is plausibly controlled by the release of CTPS from filament-like structures. Our future work will involve the live imaging to follow dynamic changes in the spatial expression of *Tg*CTPS during the invasion and egress of tachyzoites.

The dynamics of CTP synthase filament in organisms where it has been studied appears to be affected by the cell cycle, nutrient deprivation, all four nucleotides, and enzyme inhibitors. Glucose deprivation induces CTPS filament formation in yeast (Noree et al., 2010), while in mammalian cells filaments are assembled in response to deprivation of glutamine (Calise et al., 2014; Gou et al., 2014). In mammalian cells, high levels of GTP, but not CTP, are critical for disassembling the RR structures (Calise et al., 2014). In contrast, high levels of CTP stimulate the filament assembly in *E. coli* and *S. cerevisiae* (Noree et al., 2010, 2014; Barry et al., 2014). In the latter case, CTPS filaments are depolymerized by addition of ATP and UTP (Barry et al., 2014). Thus, CTP synthase might serve as a sensor of carbon sources (glucose and/or glutamine) and for the pools of each nucleotide triphosphate (Noree et al., 2010). In the case of *Tg*CTPS, limitation of glutamine resulted in an increase in filament-like structures, which was accentuated when extracellular parasites were incubated just in PBS (Figures 6E,F). It is interesting to note that glucose and glutamine serve as two major sources of carbon for the parasite (Blume et al., 2009; Nitzsche et al., 2016, 2017). Further experiments involving the parasite mutants defective in glucose and glutamine catabolism are required to determine whether the increase in CTPS structures is indeed related to the sensing of these nutrients.

DON inhibits the CTPS activity by binding to its glutaminase domain (Dion et al., 1956; Long et al., 1970). It has been studied as an anticancer drug in several Phase II clinical trials in the 1980s, because tumor cells are more susceptible to the glutamine analogs compared to normal cells. Its therapeutic usage has been abandoned due its tendency to cause nausea (Cervantes-Madrid et al., 2015), but the drug provides an excellent tool to gain the mechanistic insights of CTPS. The inhibitor has divergent effects on the localization of different CTPS enzymes. In *Cc*CTPS and *Ec*CTPS, filaments are disrupted when cells are treated with DON (Ingerson-Mahar et al., 2010; Barry et al., 2014). In contrast, DON promotes the formation of CTPS filaments in Drosophila and human cells (Carcamo et al., 2011, 2014; Chen et al., 2011), which is consistent with our observations in *T. gondii*. Analogs of glutamine such as azaserine, acivicin and DON exhibit strong anti-parasitic activities against *Plasmodium, Trypanosoma* and *Toxoplasma* (Jaffe, 1963; Hofer et al., 2001; Fox and Bzik, 2003; Fijolek et al., 2007; Nitzsche et al., 2016). We found that DON at concentrations above 5 μM caused a significant decrease in the parasite yield. Tachyzoites treated with DON were aberrantly shaped and apparently defective in endodyogeny. These results together signify a potential therapeutic repurposing of this drug to treat toxoplasmosis.

Two possible scenarios could explain the relationship of CTPS to morphological alterations observed in intracellular and extracellular parasites. In one scenario, a decrease of *Tg*CTPS activity would disrupt the membrane biogenesis because CTP is needed to form phospholipid precursors, such as CDP-diacylglycerol, CDP-ethanolamine and CDP-choline (Gupta et al., 2005; Sampels et al., 2012; Hartmann et al., 2014). Gupta and co-workers observed that an analog of choline interferes with the synthesis of phosphatidylcholine, the most abundant glycerophospholipid in the parasite. It results in a dramatic effect on growth and membrane composition concurrent with an aberrant morphology (Gupta et al., 2005). Likewise, genetic ablation of CDP-DAG synthesis exerts a detrimental effect on growth and virulence of *T. gondii* tachyzoites (Kong et al., 2017). In an alternative scenario, the ability of *Tg*CTPS to form filament-like structures and the inhibitory effect of DON on the parasite replication suggest that this protein could somehow be associated with the inner membrane complex (IMC) or with other component(s) of the parasite pellicle, the structure involved in maintaining the parasite shape. This would be consistent with a cytoskeleton-like function of CTP synthase filaments observed in some bacteria (Ingerson-Mahar et al., 2010; Liu, 2016). Our prospective work involves further characterization of filament-like structures by site-directed mutagenesis and electron microscopy to discern the mechanistic roles of CTPS structures.

*T. gondii* and other related parasites (Lim et al., 1996; Hofer et al., 2001; Yuan et al., 2005) express a single CTP synthase, in contrast to eukaryotes, such as *S. cerevisiae* (Nadkarni et al., 1995) and mammals (van Kuilenburg et al., 2000), which have at least two different CTP synthase isoforms. *TgCTPS* is expressed in all of the three archetypal *T. gondii* lineages (type I, type II and type III strains) as well as in the parasite's primary life cycle stages, bradyzoites, tachyzoites and sporulated oocysts (Fritz et al., 2012). *Tg*CTPS activity is therefore likely to be important for acute and chronic infections.

**Figure 8:**
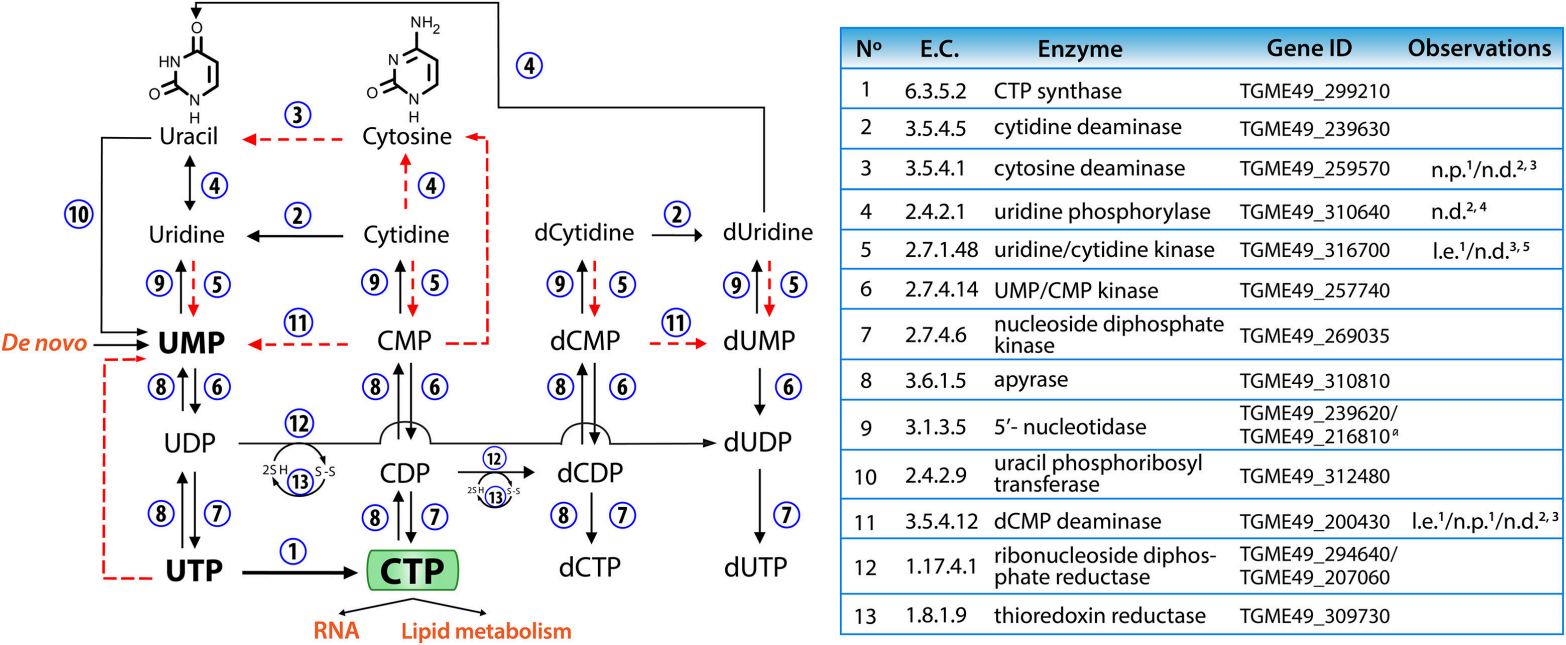
Pyrimidine salvage and interconversion reactions in *T. gondii*. The numbers next to the arrows represent the enzymes involved in each reaction, which are listed in the table. The dotted red lines show conversions that probably do not take place in tachyzoites, for the reasons given in the table (l.e.: very low expression in tachyzoites, n.d.: no activity detected, n.p.: no proteomic evidence). Data were extracted from: ^1^ToxoDB (www.toxodb.org, released 29); ^2^(Iltzsch, 1993); ^3^(Fox et al., 1999); ^4^(Fox and Bzik, 2010); ^5^(Pfefferkorn, 1978). ^*a*^ One of these genes should belong to purine metabolism. CMP, cytidine 5′-monophosphate; CDP, cytidine 5′-diphosphate; CTP, cytidine 5′-triphosphate; d-, deoxy-; UMP, uridine 5′-monophosphate; UDP, uridine 5′-diphosphate; UTP, uridine 5′-triphosphate.

In mammalian cells, CTP is obtained primarily through salvage of uridine and cytidine by uridine/cytidine kinase. In cancer cells, where the proliferation rate is increased compared to normal cells, CTP is obtained mostly via CTP synthase (van den Berg et al., 1994, 1995). In contrast, *T. gondii* has a limited pyrimidine salvage capacity especially for cytidine (Figure 8). Uridine/cytidine kinase expression levels are low in tachyzoites and enzymatic activity has not been detected (Pfefferkorn, 1978; Fox et al., 1999). Furthermore, pyrimidine phosphorylase, another salvage enzyme, was unable to use cytidine as a substrate (Iltzsch, 1993). The parasite's inability to salvage CTP to compensate for the inhibition or absence of *Tg*CTPS, supports the notion that this is an essential gene for parasite survival. Lourido and coworkers have adapted CRISPR/Cas9 technology and developed a mathematical model to determine the contribution of targeted genes to cell fitness in *T. gondii* (Sidik et al., 2016). According to this model, genes are assigned values reflecting their contribution to parasite fitness. The maximum value for a non-essential gene is 2.96 and the minimum value for an essential gene is −6.89. *TgCTPS* gene has a mean fitness score of −5.67 (Sidik et al., 2016) confirming our results that *Tg*CTPS is refractory to a genetic deletion. The physiologically essential nature of *Tg*CTPS could be exploited to design specific inhibitors of the parasite growth.

## Materials and methods

### Chemical and biological reagents

Reagents were purchased from Sigma-Aldrich unless noted otherwise. Restriction endonucleases, T4 ligase and calf intestinal alkaline phosphatase were from New England Biolabs. RNA was isolated using the Pure Link RNA Mini kit from Ambion and transcribed into first-strand cDNA using SuperScript III first-strand cDNA synthesis kit (Invitrogen). Phusion high-fidelity polymerase, dNTPs, DreamTaq DNA polymerase, GeneRuler DNA ladders, PageRuler plus prestained protein ladder, HisPur cobalt resin and the enhanced chemiluminescence (ECL) system for western blot were from Thermo Scientific. All oligonucleotides used in this study are listed in Supplementary Table 1 and were synthetized by IDT or LifeTechnologies (Invitrogen). Reagents for cell culture were from Biowest. The *pG140* and *pG152* vectors were provided by Markus Meissner (University of Glasgow, UK). The following primary antibodies were used in western blot or immunofluorescence assays: mouse or rabbit anti c-myc (monoclonal antibodies, M4439 and C3956, Sigma), anti-PentaHis antibody (Qiagen), rabbit anti-*Tg*Hsp90 (Echeverria et al., 2010) kindly provided by Sergio Angel (National University of San Martín, Argentina) and anti-*Tg*DHO (Robles Lopez et al., 2006).

### Toxoplasma and host cell culture

All transgenic and mutant parasite lines are derivatives of the RH parental strain. The Δ*ku80-hx*g*prt*^−^ (Huynh and Carruthers, 2009), Δ*ku80-TaTi* (Sheiner et al., 2011) and Δ*ku80::DiCre* (Andenmatten et al., 2013) strains were employed for genetic manipulation of the *TgCTPS* gene and were kindly provided by Vern Carruthers (University of Michigan, USA), Boris Striepen (University of Georgia, USA) and Markus Meissner (University of Glasgow, UK), respectively. Tachyzoites from all strains were propagated *in vitro* by serial passages in human foreskin fibroblasts (HFF) host cells, as previously described (Jacot et al., 2014). Briefly, parasites were routinely cultured on confluent HFF cell monolayers at a multiplicity of infection (MOI) of 3, until their complete lysis. Uninfected and infected cells were maintained in a humidified incubator (37°C, 5% CO_2_) in D10 complete medium (DMEM supplemented with 10% FBS, 2 mM L-glutamine, 1x MEM non-essential amino acids and 100 units mL^−1^ penicillin, and 100 μg mL^−1^ streptomycin). When extracellular parasites were required, they were mechanically released from the infected host cells and isolated by filtration through 3 μm filters (Nucleopore), followed by centrifugation (400 × g, 10 min, RT).

### Cloning, expression and purification of *Tg*CTPS

A cDNA Lambda Zap II library of *T. gondii* strain RH tachyzoites was obtained from the AIDS Research and Reference Reagent Program (catalog No. 1896). A phage containing the putative *TgCTPS* sequence was isolated by PCR using the method of Israel (Israel, 1993), which was packed as a phagemid and subsequently used as a template to amplify the *TgCTPS* coding sequence using Phusion high-fidelity polymerase and ORF-specific primers (Supplementary Table 1). The ORF of *TgCTPS* was cloned into *pGEM T-Easy* vector (Promega) and subcloned into *pET19b* vector (Novagen). Alternatively, cDNA template for amplification was produced from RNA extracted from 5 × 10^6^ fresh extracellular tachyzoites using the RNeasy mini kit (Qiagen). A truncated version of *Tg*CTPS lacking the first 57 residues from its N-terminus (6xHis-*Tg*CTPS-truncated) was cloned in the *pET15b* vector (Novagen). The recombinant proteins were expressed in the *E. coli* BL21-CodonPlus(DE3)RP strain. Expression conditions, purification and refolding of insoluble *Tg*CTPS by matrix assisted method are described in the Supplementary Material.

### Enzyme assays

*Tg*CTPS activity was determined by a pyruvate kinase/lactate dehydrogenase-coupled assay (Morrical et al., 1986) in a Multiskan GO spectrophotometer (Thermo Scientific). Assay parameters were fitted with the *SkanIt*T M software. The standard assay mixture consisted of 70 mM HEPES pH 8.0, 12 mM MgCl_2_, 1 mM dithiothreitol, 2 mM phosphoenolpyruvate, 0.3 mM NADH, 2 U of pre-mixed pyruvate kinase and lactate dehydrogenase (P0294, Sigma), 36.5 nM of purified *Tg*CTPS, 0.4 mM GTP, 1 mM UTP, 1 mM ATP and 4 mM glutamine in a total volume of 100 μL in a 96-well plate.

Ionic strength was adjusted to 0.2 M in all experiments using KCl. The *Tg*CTPS recombinant protein activity was measured by monitoring NADH reduction at 340 nm for 10 min at 37°C. To determine *Tg*CTPS kinetic constants, one substrate concentration was varied: L-glutamine (0–4 mM), UTP (0–3 mM), or ATP (0–3 mM), while other substrates were maintained at saturating concentrations (highest concentration in the range indicated). The effect of the allosteric activator GTP on glutamine-dependent *Tg*CTPS activity was evaluated using variable concentrations of GTP. The initial velocities for L-glutamine-dependent *Tg*CTPS activity were determined, fitted to the following equation (MacDonnell et al., 2004) and plotted (Figure 3D).

$$\begin{array}{l} \frac{v_{i}}{{\left[E\right]}_{T}}=\frac{k_{0}+\frac{k_{act}\left[GTP\right]}{K_{A}}}{1+\;\frac{\left[GTP\right]}{K_{A}}+\;{\left[\frac{\left[GTP\right]}{K_{i}}\right]}^{n}} \end{array}$$

The values of the equation parameters were: *k*_0_, 1.73 *s*^−1^; *k*_act_, 14.98 *s*^−1^; *K*_A_, 0.25 M; *K*_i_, 0.57 M and *n* = 4.69. *Tg*CTPS activity was linear with reaction time and dependent on enzyme concentration (data not shown). Kinetic parameters were determined using GraphPad Prism v6e software.

The inhibitory activity of DON was evaluated using fixed saturating concentrations of the substrates ATP (2 mM), UTP (2 mM), L-glutamine (4 mM) and GTP (0.3 mM), with varying inhibitor concentrations. The percentage of *Tg*CTPS activity retained in each DON concentration was fitted to the following equation and plotted using GraphPad Prism v6e software.

$$\begin{array}{l} Y=\frac{100}{\left(1+10^{\left(LogIC50-X\right)*HillSlope}\right)} \end{array}$$

### Immunoblot and immunofluorescense assays

SDS-PAGE and western blot analysis were performed using standard protocols (Sambrook and Russell, 2001). Briefly, proteins were separated on 12% sodium dodecyl sulfate polyacrylamide gels (SDS-PAGE) and transferred onto a nitrocellulose membrane by semi-dry transfer. Membranes were blocked in 5% non-fat milk in tris-buffered saline (TBS), 0.2% Tween, incubated with an appropriate dilution of primary antibodies (α-c-myc 1:1000, α-5XHis 1:1000 and α-*Tg*Hsp90 1:500 in blocking solution) followed by secondary antibodies conjugated to horseradish peroxidase (HRP) and detected using the ECL chemiluminescence system (GE Healthcare). Stripping of the membrane was performed using the western blot recycling kit (Alpha diagnostics international).

For IFA, confluent HFFs cells were grown on round glass coverslips within 24-well plates and then infected with *T. gondii* tachyzoites of the indicated strains at MOI of 1. After 25–30 h of incubation, the medium was removed, and the infected cells were washed twice with 1x PBS. Fixation was performed with 4% paraformaldehyde (PFA) for 10 min followed by a neutralization step in 0.1 M glycine/1x PBS for 5 min. Subsequently, cells were permeabilized in 0.2% Triton-X100/1x PBS for 20 min, followed by blocking (2–5% bovine serum albumin in 0.2% Triton-X100/1x PBS) for 30 min. The coverslips were incubated for 1 h in the primary antibody solution and washed 3 times for 5 min each in 0.2% Triton-X100/1x PBS. Afterwards, treatment with appropriate secondary antibodies diluted (1:3,000) (Alexa-Fluor 488/Alexa-Fluor 594 conjugated goat anti-mouse or anti-rabbit, Invitrogen) in blocking solution was performed for 45–60 min in the dark. Samples were mounted with DAPI-Fluoromount G (SouthernBiotech) on glass slides. Fluorescent imaging was performed using either an ApoTome Imager.Z2 microscope (Carl-Zeiss, Germany) or an Olympus FluoViewTM FV1000 unit with IX71 motorized inverted microscope (Olympus, USA).

### Functional expression of *Tg*CTPS in *S. cerevisiae*

We performed a complementation assay using *S. cerevisiae* SDO195 strain, kindly provided by Dr. George M. Carman from the Rutgers University, New Jersey (Ozier-Kalogeropoulos et al., 1994). The strain SDO195 lacks the two endogenous CTPS genes, *ura7* and *ura8*, encoding for *Sc*CTPS1 and *Sc*CTPS2, respectively (Ostrander et al., 1998; Park et al., 2003; Han et al., 2005; Chang et al., 2007). Because *ura7* and *ura8* are synthetic lethal, the growth of this strain requires the presence of a plasmid expressing a functional CTPS. In addition, it lacks beta-isopropylmalate dehydrogenase (LEU2) that catalyzes the third step in leucine biosynthesis, as well as OMP decarboxylase (URA3) catalyzing the sixth step in pyrimidine biosynthesis. The absence of LEU2 and URA3 permits the selection of transgenic strains in leucine- or uracil-free media. The SDO195 strain can grow as it harbors the plasmid pYeLac-*Sc*CTP1 along with URA3 as a selection marker (Ozier-Kalogeropoulos et al., 1994). The replacement of this plasmid with a plasmid expressing *Tg*CTPS (plasmid shuffling) was done in two steps, as described previously (Han et al., 2005). In the first step, the *Tg*CTPS coding sequence was amplified from the first-strand cDNA prepared from the tachyzoite mRNA and cloned into the pNEV-N-Leu vector (Sauer and Stolz, 1994) at the *NotI* site, which allowed the expression of *Tg*CTPS under the control of the *S. cerevisiae* ATP synthase promoter (*pPMA1*) (Sauer and Stolz, 1994). The SDO195 mutant was transformed with 500 ng of pNEV-N_*Tg*CTPS by the lithium acetate/single-stranded carrier DNA/polyethylene glycol protocol (Gietz and Woods, 2002). Additionally, we constructed a positive control plasmid, pNEV-N_*Sc*CTPS1. The empty vector was used as a negative control. Transformants were selected on plates with SC medium without leucine or uracil (Figure 2B, left). The eventual yeast strain did not require uracil for growth because they still contained *Sc*CTPS1/URA3 (Figure 2B, right). In the second step, counter-selection was performed in leuchine-free SC medium containing 1% of 5′-FOA with incubation at 30°C for 3–4 days (Figure 2C). The procedure eliminated the yeast population harboring *Sc*CTPS1/URA3 because expression of URA3 allowed 5′-FOA to be metabolized into a toxic uracil analog, which is incorporated into RNA, thereby causing death. The presence of yeast cells expressing only *Tg*CTPS/LEU2 was confirmed by growth without leucine in the presence of 5′-FOA (Figure 2C, left). These cells were auxotrophic for uracil (Figure 2C, right), since the absence of pyrimidine biosynthesis is compensated by uracil salvage.

### Expression of epitope-tagged *Tg*CTPS protein

The coding sequence of *Tg*CTPS with a c-myc tag was amplified using Pfu Ultra II fusion HS DNA polymerase (Agilent technologies) using template cDNA extracted from tachyzoites. The PCR product was digested and cloned into the *pTgGRA2-UPKO* vector using *NsiI* and *PacI* sites, which allowed a targeted insertion of *Tg*CTPS-c-myc at the *Tg*UPRT locus (Donald and Roos, 1995). The *pTgGRA2-UPKO-TgCTPS* construct was transformed into XL1-blue *E. coli* competent cells and confirmed by PCR and sequencing. The linearized construct (*ApaI*) was transfected into ≈10 × 10^6^ fresh Δ*ku80-TaTi* tachyzoites suspended in Cytomix (10 mM K_2_HPO_4_ /KH_2_PO_4_ pH 7.6, 25 mM HEPES, 120 mM KCl, 5 mM MgCl_2_, 0.15 mM CaCl_2_, 2 mM EGTA, 5 mM GSH, 5 mM ATP) using the Amaxa Nucleofector device (Lonza). Transfected parasites were selected by resistance to 5-fluoro-2′-deoxyuridine (FUDR), as described elsewhere (Donald and Roos, 1995). Parasites expressing an ectopic copy of *Tg*CTPS-c-myc protein were used for further localization experiments. For endogenous expression of *Tg*CTPS, gDNA was extracted from tachyzoites to amplify a 1.2 kb fragment of the 3′-end of the *TgCTPS* gene. As in the first construct, the reverse primer included the c-myc tag. The PCR product was digested and ligated into *pTKO-HXGPRT* vector via *XcmI* and *EcoRI* digestion. The *pTKO-HX-TgCTPS-myc* plasmid was confirmed by sequencing. The construct (10–15 μg) was linearized by *SgrAI* digestion and used to transfect tachyzoites of the *T. gondii* Δ*ku80-hx*g*prt*^−^ strain. The transfected parasites were added to the HFF monolayer cells without selection for 10–24 h. Afterwards, the standard culture medium was replaced by medium containing 25 μg mL^−1^ mycophenolic acid (MPA) and 50 μg mL^−1^ xanthine (XA) for selecting stable, transgenic parasites expressing *HXGPRT* (Donald et al., 1996). A clonal stable parasite line was isolated by limiting dilution in 96-well plates. The Δ*ku80-hx*g*prt*^−^/*Tg*CTPSc-myc/*HXGPRT* (referred to here as *RH TgCTPS_c-myc-HX*) transgenic parasite line was used for localization experiments and treatments with DON (D2141 Sigma).

### Replication and plaque assays

Replication assays were performed to evaluate the ability of *T. gondii* tachyzoites to reproduce intracellularly. Confluent HFFs cells were grown on round glass coverslips within 24-well plates and infected with fresh parasites at a MOI of 1. IFAs were performed 27–54 hpi (hours post-infection) using and anti-*Tg*Hsp90 (1:500, rabbit, Abcam) or anti-*Tg*DHO (1:800, rabbit, Strategic Biosolutions) antibodies, as described above. Three independent biological experiments were performed and the replication rates under different conditions were compared. For each assay, the number of parasites per vacuole was counted for around 100 vacuoles. Plaque assays were used to assess the overall growth fitness of the parasite strain under indicated conditions (Black and Boothroyd, 2000). HFF cell monolayers in 6-well plates were inoculated with 250–350 parasites. Plates were incubated without perturbation under indicated growth conditions. After 7 days, cells were washed with 1x PBS followed by fixation using methanol (precooled at −80°C) for 2 min and stained with crystal violet for 10 min. Three independent biological replicates were done to calculate the mean number and area of at least 50 plaques under each condition. The number of plaques is presented as a percentage value of non-treated parasites normalized to 100%. Plaques were imaged and analyzed using the ImageJ software (National Institute of Health, USA).

### DON treatment

6-diazo-5-oxo-L-norleucine (DON) (D2141, Sigma) was solubilized at 30 mM in pure water. Working dilutions for cell culture were made in DMEM from the stock solution. The concentration of DON was determined spectrophotometrically using the molar extinction coefficient at 274 nm ($\varepsilon_{1\;cm}^{1\%}=683$) (Dion et al., 1956).

Parasites expressing *Tg*CTPS at endogenous levels (*RH TgCTPS_c-myc-HX* strain) were allowed to infect confluent HFF cells in the presence of variable concentrations of DON ranging from 2 to 20 μM during 6–54 h at 37°C. Extracellular parasites were treated with variable concentrations of DON (2–20 μM) in complete medium for 3–6 h at 37°C. The effect of DON in the *Tg*CTPS polymerization in intracellular and extracellular parasites was evaluated by IFA, while its effect on parasite fitness was evaluated by replication and plaque assays. We observed no apparent effect on HFF cells cultured in 20 μM DON.

### Genetic manipulation at the *Tg*CTPS locus

For the gene knockdown, a fragment of 1.2 kb of the 3′-end of the *TgCTPS* genomic sequence was amplified and cloned into the *pG152* vector by ligation independent cloning (Stols et al., 2002). Linearized plasmid (*XhoI*, 10–15 μg) was used to transfect tachyzoites from the Δ*ku80::DiCre* strain. Transgenic parasites were selected for *HXGPRT* expression as described above. The attempt to generate a conditional mutant by Cre recombinase activity was performed using a gene-swap strategy (Andenmatten et al., 2013). The construct was generated in three sequential steps as follows: the *TgCTPS* 5′-UTR (1.5 kb) and 3'UTR (1 kb) were amplified from *T. gondii* gDNA and inserted into the *p*5*RT*70loxP*-KillerRed*loxP*-YFP-HXGPRT* plasmid (Andenmatten et al., 2013) between *ApaI* and *SacI* sites, respectively. The *TgCTPS* ORF plus a c-myc tag was amplified from *T. gondii* cDNA, digested with *MfeI* and *PacI* and cloned into the vector using *EcoRI* and *PacI* restriction sites to replace the *killerRed* ORF. The resulting *T*g*CTP*5′UTR-*p*5*RT*70–loxP*T*g*CTP*loxP-*YFP-HXGPRT*-*T*g*CTP*3′UTR vector was linearized via *ScaI* and transfected into Δ*ku80::DiCre* recipient strain. Transgenic parasites were selected by the treatment with XA and MPA. The *TgCTPS* conditional mutant was constructed using the Tet-TA system (Meissner, 2001, 2002). In the first step, the *TgCTPS* ORF including a C-terminal c-myc tag was cloned into the tetracycline-regulated expression vector (pNTP3TetO7Sag1) using the *NcoI* and *PacI* restriction sites. The resulting plasmid *pNTP3TetO7Sag1-TgCTPS-c-myc*, which is a UPKO-based vector, allowed the insertion of a tetracycline-regulatable copy of the C-terminally c-myc-tagged *Tg*CTPS at the *Tg*UPRT locus via double homologous recombination. The construct was linearized using *ApaI* and *NcoI* restriction enzymes and transfected into the Δ*ku80-TaTi* strain. Transgenic parasites were selected with 5 μM FUDR followed by isolation of a clonal line by limited dilution. The tetracycline regulation of *Tg*CTPS-c-myc was confirmed by IFA, comparing parasites cultured without anhydrotetracycline (ATc) *versus* parasites cultured for 40 h in medium containing 0.5 μM ATc. In the second step, the 5′UTR (1 kb) and 3′UTR (1.2 kb) fragments of the *TgCTPS* gene were amplified from gDNA and cloned into the *pTub8CAT* vector at the *ApaI* and *XhoI/XbaI* sites, respectively, flanking the chloramphenicol acetyltransferase (CAT) resistance cassette. The construct was linearized with *XbaI* and transfected into the parasites obtained in step one (parasites where the *Tg*UPRT locus was replaced by *Tg*CTPS-c-myc). Stable parasites were selected with chloramphenicol as previously described (Kim et al., 1993) and the appropriate integration of the *T*g*CTPS*5′UTR-*CAT*-*T*g*CTPS*3′UTR plasmid was verified by recombination-specific PCR.

## Author contributions

HN-O, BZ, and NG designed the experiments. HN-O and AL performed the experiments. HN-O, NG, and BZ analyzed the experiments and wrote the paper. All authors reviewed the results and approved the final version of the manuscript.

### Conflict of interest statement

The authors declare that the research was conducted in the absence of any commercial or financial relationships that could be construed as a potential conflict of interest.
