# Supplementary material for: A CTP Synthase Undergoing Stage-Specific Spatial Expression Is Essential for the Survival of the Intracellular Parasite Toxoplasma gondii
Source: Front Cell Infect Microbiol. 2018 Mar 22;8:83. doi: 10.3389/fcimb.2018.00083 (PMC5874296; doi:10.3389/fcimb.2018.00083)
Supplement: Supplementary file 1 [file DataSheet1.pdf]

Supplementary Material

A CTP synthase undergoing stage specific spatial expression is essential for the survival of the intracellular parasite *Toxoplasma gondii*

Heidy Y. Narvaez-Ortiz[1], Andrea J. Lopez[1], Nishith Gupta[2*], Barbara H. Zimmermann[1*]

*** Correspondence:** gupta.nishith@staff.hu-berlin.de, bazimmer@uniandes.edu.co

Supplementary Data

Purification of *Tg*CTPS from inclusion bodies

Standard induction was as follows, cells were grown to an OD_{600} of 0.3 at 37 ºC, cooled to RT, incubated until OD_{600} of 0.5, and then induced with 1 mM IPTG for 3 h. Cells were harvested by centrifugation (6,000 × *g,* 20 min, 4 °C) and a pellet from 1 L of bacterial cell culture was resuspended in 20 mL of buffer A (50 mM sodium phosphate buffer pH 7.4, 500 mM NaCl and 10% glycerol) containing 5 mM imidazole, 1 mM phenylmethanesulfonyl fluoride (PMSF) and 1 mM benzamidine. Lysozyme (1 mg/mL) was added to the cell suspension and it was incubated on ice for 2 h. Cells were disrupted by sonication on ice (30 cycles of 20 sec each, output control setting of 8 and 100% duty cycle) using a 250 Analog Sonifier (Branson). The two protease inhibitors were added to the suspension three times during the sonication process reaching a final concentration of 3 mM each. After sonication, insoluble and soluble fractions were separated by centrifugation (10,000 × *g*, 1 h, 4 °C). Because most of the *Tg*CTPS full length protein was insoluble under native conditions, the pellet was reserved for recovering protein from IBs. To purify N-terminally truncated *Tg*CTPS under native conditions, the clarified cell lysate was loaded onto 1 mL of a pre-equilibrated Co^{2+} affinity column. The column was washed with 10 volumes of buffer A containing 35 mM imidazole. *Tg*CTPS was eluted with 1.5 mL of buffer A containing 300 mM imidazole. The protein-containing fractions were concentrated using a centrifugal filter unit (Millipore, 30 kDa or 100 kDa MWCO). The concentration of purified protein was measured by the Bradford assay (BioRad) with bovine serum albumin as the standard.

The pellet obtained after cell disruption was treated with the denaturing agent urea, to recover *Tg*CTPS from IBs. First, the pellet was washed 3x in buffer A containing 0.5% Triton X100. Washed pellet was resuspended in buffer A containing 6 M urea, and incubated on ice for 2 h with gently shaking followed by centrifugation (10,000 × *g*, 1 h, 4 °C). The supernatant was loaded onto 1 mL of a pre-equilibrated Co^{2+} affinity column that was maintained under denaturing conditions, i.e. buffers containing 6 M urea. The column was washed with 10 volumes each of buffer B (buffer A, 0.5 mM oxidized glutathione (GSSG), 5 mM reduced glutathione (GSH), 35 mM imidazole) containing descending concentrations of urea from 6 M to 0 M (10 volumes containing 5 M urea, then 10 volumes containing 4 M urea, etc.). The refolded *Tg*CTPS protein was eluted with 1 mL of buffer B containing 400 mM imidazole. A final elution of 1.5 mL with buffer B containing 500 mM imidazole was performed in order to remove all the remaining *Tg*CTPS from the column.

.

1 Supplementary Figures and Table

```
                                                                             **  *  ***                       *** * * *   *
Tgon       1  MNAASRAASCPRLDAEFPVPCSLNSPQLFCGHNIFKMATGADSLEFVGNHSPAGGKTKYIVVTGGTMSGLGKGTTISS-LGVTLKALGVHVTAIEIDPYLNVDAGTMSPY
Pfal       1                                             MDSVDDEKP---IIKYIIVTGGNMSGLGKGTAAMSRYGCIIVNKKHFINNNKVDPYLNIDAGTMSPY
Lmaj       1                              MEERKTASGSAYYLPSPLLLSQNQGEERVKFVVVSGGVCSSLGKGVTTSA-IGALLHASGYRVCSIKIDPYINIDAGLMSPY
ScerI      1                                                          MKYVVVSGGVISGIGKGVLASS-TGMLMKTLGLKVTSIKIDPYMNIDAGTMSPL
ScerII     1                                                          MKYVVVSGGVISGIGKGVLASS-TGMLLKTLGLKVTSIKIDPYMNIDAGTMSPL
HsapI      1                                                          MKYILVTGGVISGIGKGIIASS-VGTILKSCGLHVTSIKIDPYINIDAGTFSPY
HsapII     1                                                          MKYILVTGGVISGIGKGIIASS-IGTILKSCGLRVTAIKIDPYINIDAGTFSPY
DmegC      1                                                          MKYILVTGGVISGVGKGVIASS-FGTLLKSCGLDVTSIKIDPYINIDAGTFSPY
Tther      1                                               MNGSADAGPRPRKYVFITGGVVSSLGKGILTSS-LGALLRARGYRVTAIKIDPYVNVDAGTMRPY
Ecol       1                                                        MTTNYIFVTGGVVSSLGKGIAAAS-LAAILEARGLNVTIMKLDPYINVDPGTMSPI
Ccre       1                                                         MTRYIFITGGVVSSLGKGLASAA-LGALLQARGYKVRLRKLDPYLNVDPGTMSPY

               ***  *  ** * *** * ***          * * *  *      ** *  *  ***  *  *                                    * *** **
Tgon     110  EHGEVYVLEDGGEVDLDLGNYERFLDITLTRDHNLTSGKVYQKVIQEERKGSYLGKTVQVVPQVTDAIQAWIARVAEQ---------PVDGHYEAPQVCLIEVGGTVGDI
Pfal      65  EHGEVYVLEDGGEVDLDLGNYERFLNIRLTYKNNITSGKIYEEVIKKERKGEYLGKTVQVVPHVTDAIQKWIKDVIDENIKKMKKEYNIDSFNKIPCMCLIEVGGTVGDI
Lmaj      82  EHGEVYVLEDGGEADLDLGNYERWMSVHLTRDHNITTGKMYQKLLTKERAGGFLGKTVQLVPHFTNEVVNHIFKICQT---------PMGGSNKRPEICMIELGGTVGDM
ScerI     54  EHGECFVLDDGGETDLDLGNYERYLGVTLTKDHNITTGKIYSHVIAKERKGDYLGKTVQIVPHLTNAIQDWIERVAKI---------PVDDTGMEPDVCIIELGGTVGDI
ScerII    54  EHGECFVLDDGGETDLDLGNYERYLGITLSRDHNITTGKIYSQLISRERRGDYLGKTVQIVPHLTNAIQDWIQRVSKI---------PVDDTGLEPDVCIIELGGTVGDI
HsapI     54  EHGEVFVLDDGGEVDLDLGNYERFLDIRLTKDNNLTTGKIYQYVINKERKGDYLGKTVQVVPHITDAIQEWVMRQALI---------PVDEDGLEPQVCVIELGGTVGDI
HsapII    54  EHGEVFVLNDGGEVDLDLGNYERFLDINLYKDNNITTGKIYQHVINKERRGDYLGKTVQVVPHITDAVQEWVMNQAKV---------PVDGNKEEPQICVIELGGTIGDI
DmegC     54  EHGEVYVLDDGAEVDLDLGNYERFLDVTLHRDNNITTGKIYKLVIEKERTGEYLGKTVQVVPHITDAIQEWVERVAQT---------PVQGSS-KPQVCIVELGGTIGDI
Tther     65  EHGEVFVTADGAETDLDIGHYERFLDMDLSRGNNLTTGQVYLSVIQKERRGEYLSQTVQVIPHITDEIKERIRKVAE---------------EQKAEIVVVEVGGTVGDI
Ecol      56  QHGEVFVTEDGAETDLDLGHYERFIRTKMSRRNNFTTGRIYSDVLRKERRGDYLGATVQVIPHITNAIKERVLE-----------------GGEGHDVVLVEIGGTVGDI
Ccre      55  QHGEVFVTDDGAETDLDLGHYERFTGVSATKADNITTGQIYKTIIEKERRGDYLGATVQVIPHVTNEIKDFVLSPAMD-----------ETGKKPVDFVLVEIGGTVGDI

              *     **                *    *        ******            **    *           *      *              *  *
Tgon     211  ESAVYLEALQQFSRRVGRENLCLCHVSYVPCIG--GEQKTKPTQHGVKELRMAGLSPDMIFCRCETMLSEAARGKIALFTQVLPEHVISVHDVANTYSVPLVLDSQNVAH
Pfal     175  ESAVYLEALQQLINNLNNDDVCLCHLSYVPIIGNLREQKTKPTQHSVKILREAGLKPDFIFCRCEEPLTQEAIKKIALFSQVKNEHVISLHDTSNVYKVPLILDKQNVCM
Lmaj     183  ESQPFVEALRRLRYSILPEDFCLVHTTYLPVFG--GTQKTKPTQHSCRALLSLGLQPDFLVCRSEQPLAADVKAKLSDQCGVQSKDIIGAPDVNCLYQIPVKFVDQGLID
ScerI    155  ESAPFVEALRQFQFKVGKENFALIHVSLVP--VIHGEQKTKPTQAAIKGLRSLGLVPDMIACRCSETLDKPTIDKIAMFCHVGPEQVVNVHDVNSTYHVPLLLLEQKMID
ScerII   155  ESAPFVEALRQFQFEVGRENFALIHVSLVP--VIHGEQKTKPTQAAIKDLRLLGLIPDMIACRCSEELNRSTIDKIAMFCHVGPEQVVNVHDVNSTYHVPLLLLKQHMID
HsapI    155  ESMPFIEAFRQFQFKVKRENFCNIHVSLVPQPSSTGEQKTKPTQNSVRELRGLGLSPDLVVCRCSNPLDTSVKEKISMFCHVEPEQVICVHDVSSIYRVPLLLEEQGVVD
HsapII   155  EGMPFVEAFRQFQFKAKRENFCNIHVSLVPQLSATGEQKTKPTQNSVRALRGLGLSPDLIVCRSSTPIEMAVKEKISMFCHVNPEQVICIHDVSSTYRVPVLLEEQSIVK
DmegC    154  EGMPFVEAFRQFQFRVKRENFCLAHVSLVPLPKATGEPKTKPTQSSVRELRGCGLSPDLIVCRSEKPIGLEVKEKISNFCHVGPDQVICIHDLNSIYHVPLLMEQNGVIE
Tther    160  ESLPFLEAIRQFRFDEGEGNTLYLHLTLVPYLETSEEFKTKPTQHSVATLRGVGIQPDILVLRSARPVPEEVRRKVALFTNVRPGHVFSSPTVEHLYEVPLLLEEQGLGR
Ecol     149  ESLPFLEAIRQMAVEIGREHTLFMHLTLVPYMAASGEVKTKPTQHSVKELLSIGIQPDILICRSDRAVPANERAKIALFCNVPEKAVISLKDVDSIYKIPGLLKSQGLDD
Ccre     154  EGLPFFEAIRQLRQDLPRQQSCYIHLTLLPFIKTAGEMKTKPTQHSVKELRSIGIQPDILLCRCEQEIPAEEKRKIAQFCNVRPSAVIQAMDSSSIYAVPIDYHEQGLDA

                                                                    ***     * * *      *
Tgon     319  SICKRLRLDVATPASPASRPGAPLYKTPISMKKWRLMADRLTSPAES-VKIGIVGKYTG-LADSYLSVVKALQHGAMEANVRLELVWIESSDLEEPPRE-----------
Pfal     285  NVLKKLNLENVVLNN-------KLQISPYSFNIWKQLADRYESSSEQ-VVIGIVGKYTA-SNDTYLSIISSLVHACLECGFKLVIKYINSSHLSLKQKKQKKNMDWKKKA
Lmaj     291  RLLHKLRLRPN-----------MPPTDAPTYQTFKKFSDILQNPNNQKVRIAFVGKYVTGGSDAYFSVLQCFEHCQLALGINVEVFYLESEQLEGENAE-----------
ScerI    263  YLHARLKLDEISLTE------EEKQRGLELLSKWKATTGNFDESMET-VKIRLVGKYTN-LKDSYLSVIKALEHSSMKCRRKLDIKWVEATDLEPEAQE-----------
ScerII   263  YLHSRLKLGEVPLTL------EDKERGSQLLTNWENMTKNLDDSDDV-VKIALVGKYTN-LKDSYLSVTKSLEHASMKCRRQLEILWVEASNLEPETQE-----------
HsapI    265  YFLRRLDLP------------IERQP-RKMLMKWKEMADRYDRLLET-CSIALVGKYTK-FSDSYASVIKALEHSALAINHKLEIKYIDSADLEPITSQ-----------
HsapII   265  YFKERLHLP------------IGDSA-SNLLFKWRNMADRYERLQKI-CSIALVGKYTK-LRDCYASVFKALEHSALAINHKLNLMYIDSIDLEKITET-----------
DmegC    264  YLNERLQLN------------IDMSKRTKCLQQWRDLARRTETVRRE-VCIAVVGKYTK-FTDSYASVVKALQHAALAVNRKLELVFIESCLLEEETLH-----------
Tther    270  AVERALGLE----------------AVIPNLSFWQEAVRVLKHPERT-VKIAIAGKYVK-MPDAYLSLLEALRHAGIKNRARVEVKWVDAESLEAAD-------------
Ecol     259  YICKRFSLN----------------CPEANLSEWEQVIFEEANPVSE-VTIGMVGKYIE-LPDAYKSVIEALKHGGLKNRVSVNIKLIDSQDVETR--------------
Ccre     264  EVLDVFGMSD---------------APAPDLSRWKTIDQTVQHPDGE-VTIAVVGKYTV-LKDAYKSLIEALHHGGLANKVKVNLDWVESETFEGDEGA-----------

                                                                  ****  **  *        *    *  * * * * *     *           *
Tgon     416  -----------------------SAASNGDSATPARFAAAWEALRSVAGVVCPGGFGDRGILGKALSSRFCRENKVPYLGICLGMQTAVIDFARSVLNLADANSEEFDKT
Pfal     386  RKYSYENFHENSSTEKIIFVDDTTSDEDYDKKRRIKYEKAWETLKSVDGVLVPGGFGTRGIEGKYLSSKYCRLHNIPYLGICLGMQIAVIDVAREYLNVN-ANSEEFEDT
Lmaj     379  --------------------------------------EAEAALLECDGIFVPGGFGVRGIDGKVNAVRLAREHNIPYFGVCLGMQVALIEFARSQLGWEDANSEEFDAS
ScerI    354  -------------------------------SNKTKFHEAWNMVSTADGILIPGGFGVRGTEGMVLAARWARENHIPFLGVCLGLQIATIEFTRSSLGRKDSHSAEFYPD
ScerII   354  -------------------------------VDKNKFHDSWNKLSSADGILVPGGFGTRGIEGMILAAKWARESGVPFLGVCLGLQVAAIEFARNVIGRPNSSSTEFLDE
HsapI    349  -------------------------------EEPVRYHEAWQKLCSAHGVLVPGGFGVRGTEGKIQAIAWARNQKKPFLGVCLGMQLAVVEFSRNVLGWQDANSTEFDPT
HsapII   349  -------------------------------EDPVKFHEAWQKLCKADGILVPGGFGIRGTLGKLQAISWARTKKIPFLGVCLGMQLAVIEFARNCLNLKDADSTEFRPN
DmegC    349  -------------------------------SEPSKYHKEWQKLCDSHGILVPGGFGSRGMEGKIRACQWARENQKPLLGICLGLQAAVIEFARNKLGLKDANTTEIDPN
Tther    349  ---------------------------------------LDEAFRDVSGILVPGGFGVRGIEGKVRAAQYARERKIPYLGICLGLQIAVIEFARNVAGLKGANSTEFDPH
Ecol     337  ---------------------------------------GVEILKGLDAILVPGGFGYRGVEGMITTARFARENNIPYLGICLGMQVALIDYARHVANMENANSTEFVPD
Ccre     346  ---------------------------------------AAARLENAHAIMVPGGFGERGAEGKIRAAQFARERKVPYFGICFGMQMAVIETLRNVAGIKDASSSEFGPT
                                                                                             ★

Tgon     503  ---------------------------------------CKHH----------------------------------------------------VVVSMPEHSAEHGQE
Pfal     495  TEMQYTSHENNIADSDNYNKDDNNNDNNQHAKKNPNVTNCTYNEKEKKEKKDKKENIINLENLENVENVENVENVENVNKTSVHINEICSQDTTASCISIKTNSSHNPKE
Lmaj     451  -----------------------------------------------------------------------------------------------STRQVVRIMDADRER
ScerI    433  --------------------------------------------------------------------------------------------IDEKNHVVVFMPEIDKET
ScerII   433  TL------------------------------------------------------------------------------------------LAPEDPSSHIHAEIDKEH
HsapI    428  ----------------------------------------------------------------------------------------------TSHPVVVDMPEHNPGQ
HsapII   428  ----------------------------------------------------------------------------------------------APVPLVIDMPEHNPGN
DmegC    428  ----------------------------------------------------------------------------------------------TANALVIDMPEHHTGQ
Tther    420  TPH-------------------------------------------------------------------------------------------PVIDLMPEQLEVEG--
Ecol     408  CKY-------------------------------------------------------------------------------------------PVVALITEWRDENGNV
Ccre     417  DR--------------------------------------------------------------------------------------------PVVGLMTEWIKGNEMV
```

```
Tgon     522  LG------------------------------------------------------------------------------------------------------------
Pfal     605  LASYTERQKHDNINNLKKEIHFDDNISTAIINDDINIENSIYSKYQDTYMTDKNTYDDTDPMFDGKEYLKSQSLILREKCIEDIPKKLSEESIKEKIKLIGIESYYKSIE
Lmaj     466  MG------------------------------------------------------------------------------------------------------------
ScerI    451  MG------------------------------------------------------------------------------------------------------------
ScerII   453  MG------------------------------------------------------------------------------------------------------------
HsapI    444  MG------------------------------------------------------------------------------------------------------------
HsapII   444  LG------------------------------------------------------------------------------------------------------------
DmegC    444  LG------------------------------------------------------------------------------------------------------------
Tther    437  --------------------------------------------------------------------------------------------------------------
Ecol     427  EVRSEK--------------------------------------------------------------------------------------------------------
Ccre     435  QRRAN---------------------------------------------------------------------------------------------------------

                                     ****                  *       ****** *                               *  *          * **
Tgon     524  ---------------------GTMRLGKRATILRDSK--SLAARLYDGKPVIDERHRHRYEVNPSVVGSMEAKGFMFVGQDE----RGQ-RMEVAELRDHPFFLCVQYHP
Pfal     715  EIDNNNVIISMSEFKGDDNKGGTMRLGVKQSKIIDKD--SLTYKAYDEELYIYERHRHRYEINTKYVPLLEAVGLTFVAKDI----HSVPRMEICEIKNLDFYVGVQFHP
Lmaj     468  ---------------------ANMHLGAREVHIVEPH--SRMSTIYSGAKVVLERHRHRYEAHGKYLNHLRKQGLVISAVSDP-DAGENLRVEAIENPSLKFFLAVQFHP
ScerI    453  ---------------------GSMRLGLRPTFFQNETEWSQIKKLYGDVSEVHERHRHRYEINPKMVDELENNGLIFVGKDD----TGK-RCEILELKNHPYYIATQYHP
ScerII   455  ---------------------GTMRLGLRPTIFQPNSEWSNIRKLYGEVNEVHERHRHRYEINPKIVNDMESRGFIFVGKDE----TGQ-RCEIFELKGHPYYVGTQYHP
HsapI    446  ---------------------GTMRLGKRRTLFQTKN--SVMRKLYGDADYLEERHRHRFEVNPVWKKCLEEQGLKFVGQDV----EGE-RMEIVELEDHPFFVGVQYHP
HsapII   446  ---------------------GTMRLGIRRTVFKTEN--SILRKLYGDVPFIEERHRHRFEVNPNLIKQFEQNDLSFVGQDV----DGD-RMEIIELANHPYFVGVQFHP
DmegC    446  ---------------------GTMRLGKRITVFSDGP--SVIRQLYGNPKSVQERHRHRYEVNPKYVHLLEEQGMRFVGTDV----DKT-RMEIIELSGHPYFVATQYHP
Tther    437  -------------------LGGTMRLGDWPMRIKPGT---LLHRLYGKEE-VLERHRHRYEVNPLYVDGLERAGLVVSATTPGMRGRGAGLVEAIELKDHPFFLGLQSHP
Ecol     433  -----------------SDLGGTMRLGAQQCQLVDDS---LVRQLYNAPT-IVERHRHRYEVNNMLLKQIEDAGLRVAGRSG-----DDQLVEIIEVPNHPWFVACQFHP
Ccre     440  -----------------DDLGGTMRLGAYDAVLTAGS---KVAQIYGGTE-ISERHRHRYEVNIGYVHLMEEAGLKLTGRSP-----NGVLPEIVERDDHPWFIGVQYHP

              *  *
Tgon     606  EFQSRPLKPSPPFLGLVLAAAGKLEARFKRYGGFLKSG-----AVYEEVESA
Pfal     819  EFTSRPFKSNPLFLAFVLASKKKLKDRLNKYGNKLCSG-----ILYK
Lmaj     554  EFVSTPLDPSPPYLAFFAAAAGKEVNWPAECTARRLPASA
ScerI    537  EYTSKVLDPSKPFLGLVAASAGILQDVIEG-KYDLEAG--------ENKFNF
ScerII   539  EYTSKVLEPSRPFWGLVAQLR-HTCEVIKD-INLSEGN--------ENE
HsapI    528  EFLSRPIKPSPPYFGLLLASVGRLSHYLQK-GCRLSPR---DTYSDRSGSSS----PDSEITELKFPSINHD
HsapII   528  EFSSRPMKPSPPYLGLLLAATGNLNAYLQQ-GCKLSSS---DRYSDASDDSF----SEPRIAELEIS
DmegC    528  EYLSRPLKPSPPFLGLILASVDRLNQYIQR-GCRLSPRQLSDASSDEEDSVVGLAGATKSLSSLKIPITPTNGISKSCNGSISTSDSEGACGGVDPTNGHK
Tther    524  EFKSRPMRPSPPFVGFVEAALAYQERA
Ecol     517  EFTSTPRDGHPLFAGFVKAASEFQKRQAK
Ccre     524  ELKSRPFAPHPLFASFIAAAKEHGRLV
```

Supplementary Figure 1. Multiple sequence alignment of CTP synthases from different organisms. Conserved residues in all proteins are shown in bold and indicated by asterisks above the sequences. Residues conserved in ≥85% of CTP synthases are colored by chemical property according to the STRAP server. The highly conserved regions of the synthase domain, which are required for UTP, ATP and CTP binding, are underlined in purple. The linker between the synthase and glutaminase domain is underlined in green. The catalytic triad (Cys-His-Glu) is indicated by ★ below the sequences. The N-terminal extension of *Tg*CTPS is indicated by a pink box. The multiple sequence alignment was performed using the MAFFT algorithm (Katoh *et al*., 2002). The alignment figure was produced using the STRAP server (Gille *et al*., 2014). The proteins included in the alignment are, in descending order, as follows: *Toxoplasma gondii,* Tgon, JN847214.1; *Plasmodium falciparum*, Pfal, AAC36385.1; *Leshmania major*, Lmaj, XP_001682814.1; *Saccharomyces cerevisiae* I, ScerI, CAA37941.1; *S. cerevisiae* II, ScerII, CAA48277.1; *Homo sapiens* I, HsapI, NP_001896; *H. sapiens* II, HsapII, NP_062831; *Drosophila melanogaster* isoform C, DmegC, NP_730023.1; *Escherichia coli*, Ecol, ACB03894.1; *Thermus thermophilus*, Tther, YP_144732.1. *Caulobacter crescentus*, Ccre, ENZ83858.1.

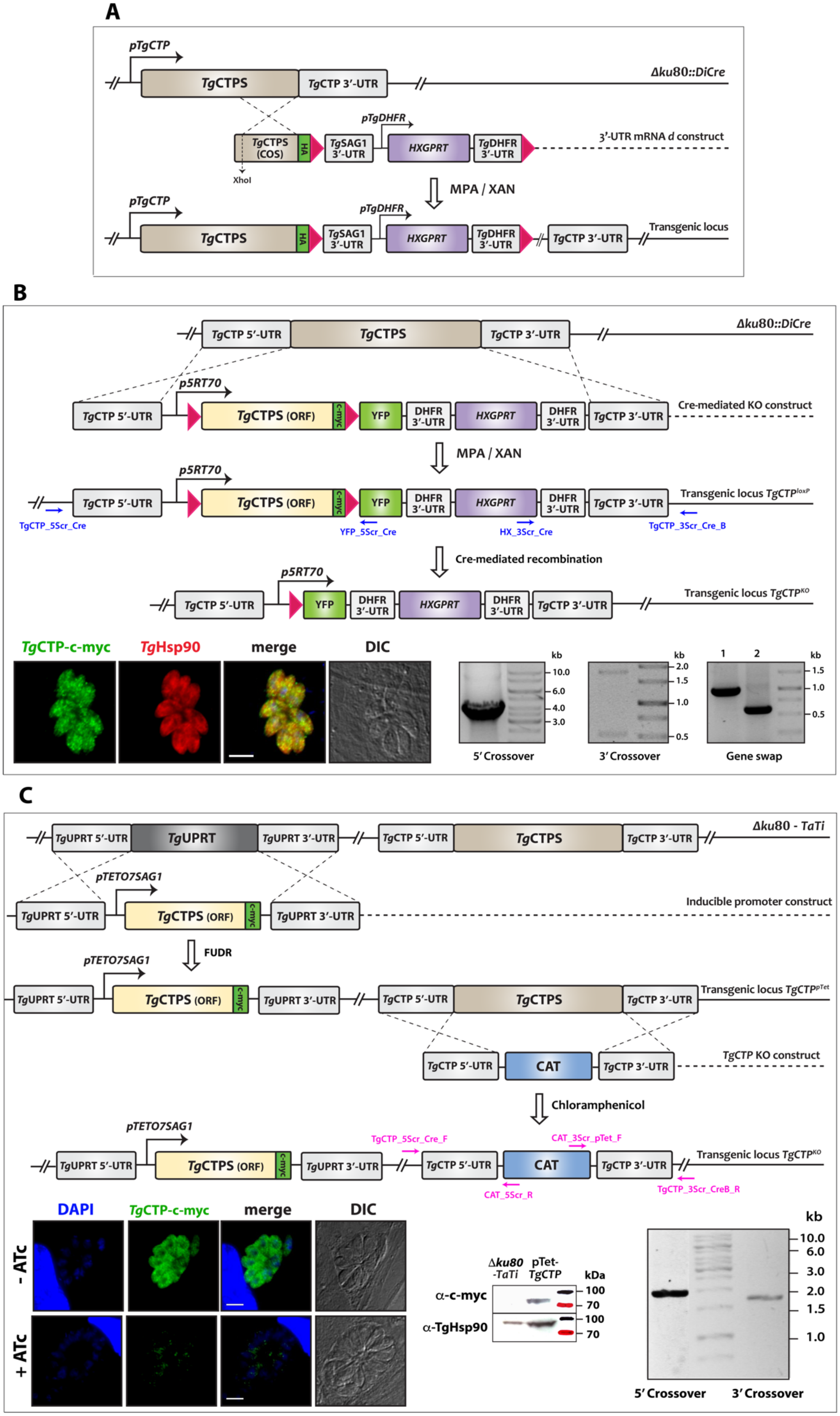
A
pTgCTP
TgCTPS
TgCTP 3′-UTR
Δku80::DiCre
pTgDHFR
TgCTPS (COS)
HA
TgSAG1 3′-UTR
HXGPRT
TgDHFR 3′-UTR
3′-UTR mRNA d construct
XhoI
MPA / XAN
Transgenic locus
B
TgCTP 5′-UTR
p5RT70
TgCTPS (ORF)
c-myc
YFP
DHFR 3′-UTR
Cre-mediated KO construct
Transgenic locus TgCTP^loxP
TgCTP_5Scr_Cre
YFP_5Scr_Cre
HX_3Scr_Cre
TgCTP_3Scr_Cre_B
Cre-mediated recombination
Transgenic locus TgCTP^KO
TgCTP-c-myc
TgHsp90
merge
DIC
kb
10.0
6.0
4.0
3.0
2.0
1.5
1.0
0.5
5′ Crossover
3′ Crossover
Gene swap
C
Δku80 - TaTi
TgUPRT 5′-UTR
TgUPRT
TgUPRT 3′-UTR
pTETO7SAG1
Inducible promoter construct
FUDR
Transgenic locus TgCTP^pTet
CAT
TgCTP KO construct
Chloramphenicol
TgCTP_5Scr_Cre_F
CAT_3Scr_pTet_F
CAT_5Scr_R
TgCTP_3Scr_CreB_R
DAPI
- ATc
+ ATc
Δku80 -TaTi
pTet-TgCTP
kDa
α-c-myc
α-TgHsp90
100
70
5′ Crossover
3′ Crossover

Supplementary Figure 2. Scheme illustrating *Tg*CTPS knockout and conditional knockout strategies. **(A)** 3'UTR destabilization of *TgCTPS* mRNA. The loxP sites are represented by red triangles. **(B)** The Cre recombinase-mediated knockout of the *TgCTPS* gene via double homologous crossover. In the gene swap strategy the *TgCTPS* endogenous locus was replaced by the cassette labeled Cre-mediated KO construct in the *Δku80::DiCre* strain. The *TgCTPS* cDNA was cloned between two loxP sites and followed by yellow fluorescence protein (YFP) and the selection marker *HXGPRT*. Although, the recombination-specific PCR identified weak amplifications in the pool of transfected parasites, only single crossover events at the 5'- or 3'-end, were observed in individual clones. **(C)** Conditional knockout of *TgCTPS*. The inducible promoter construct was transfected in parasites from the *Δku80-TaTi* strain and selected with FUDR to obtain the transgenic locus *TgCTP* ^{pTet} containing the endogenous and the ATc inducible copy of *TgCTPS*. The 5'- and 3'-recombination-specific PCRs identified amplifications in the pool of transfected parasites, but a double crossover was never observed in individual clones.

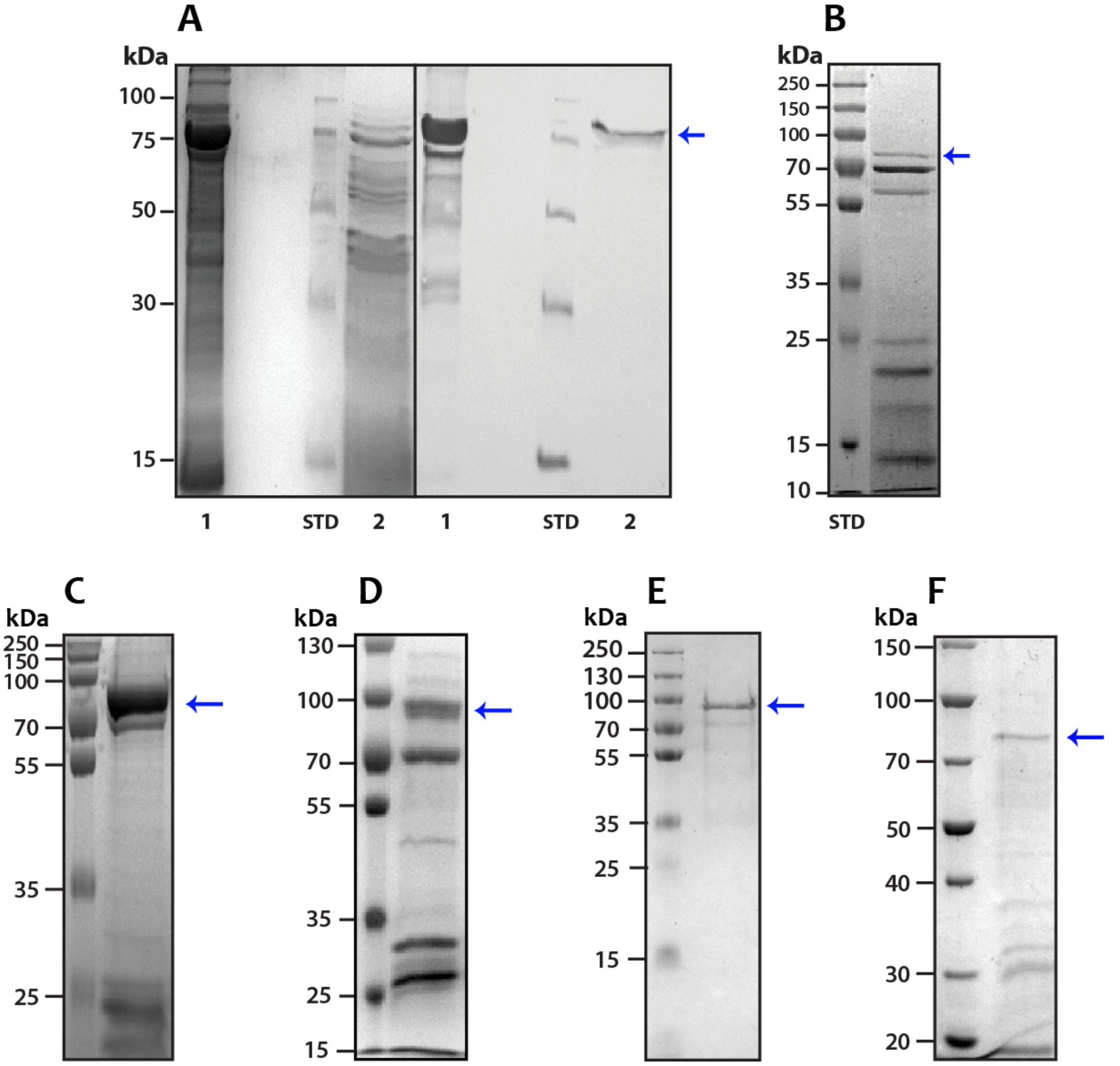

Supplementary Figure 3. Recombinant expression of *Tg*CTPS. (A) Under standard conditions, a small amount of soluble recombinant protein was observed by SDS-PAGE (left panel), and was confirmed by western blot using anti-His tag antibodies (right panel). *Lane 1,* pellet obtained after centrifugation of sonicated induced cells; *Lane 2,* supernatant obtained after centrifugation of sonicated induced cells. *STD*, 6xHis protein ladder (Qiagen). **(B)** *Tg*CTPS obtained under standard expression conditions, and concentrated with a centrifugal filter (100 kDa, MWCO, Millipore). The arrow indicates the band corresponding to *Tg*CTPS, which was confirmed by western blot (data not shown). **C – F.** SDS-PAGE of *Tg*CTPS produced under different conditions. In all cases, only one elution fraction obtained afte9r affinity chromatography using Co^{+2} resin is shown. **(C)** Induction of expression of the full length *Tg*CTPS in TB media at low temperature in the presence of low concentrations of IPTG as described previously (Steeves and Bearne, 2011). (**D)** Full length *Tg*CTPS produced by autoinduction in minimal medium (Studier, 2005). (**E)** Full length *Tg*CTPS protein recovered from inclusion bodies using urea in the presence of reduced/oxidized glutathione (10 GSH/1 GSSG). (**F)** Truncated *Tg*CTPS obtained after purification by affinity chromatography using

Co^{+2} resin. The blue arrows indicate the bands corresponding to *Tg*CTPS, which were confirmed by western blot (data not shown). **B-E** PageRuler plus prestained protein ladder (ThermoScientific) was used as a standard. **F.** PageRuler unstained broad range protein ladder (ThermoScientific) was used as the standard.

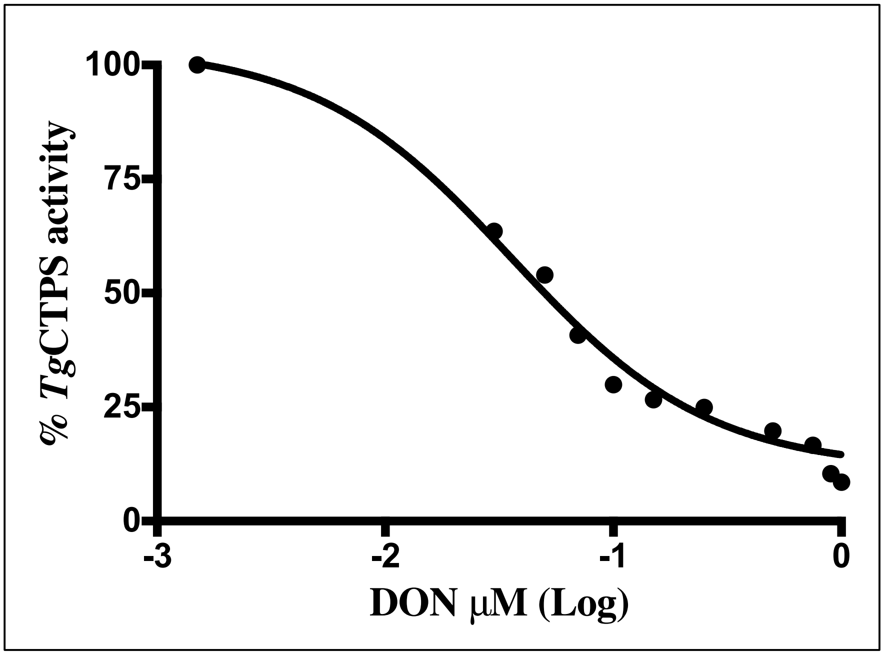

Supplementary Figure 4. Dose-response inhibition curve of DON for *Tg*CTPS. The rate of CTP formation was measured in the presence of a saturating concentration of L-Gln (4 mM), ATP (1 mM), UTP (1 mM) and GTP (0.4 mM). The curve shown was generated from a nonlinear regression fit of the data to log(inhibitor) vs. response using the GraphPad Prism version 6.0e.

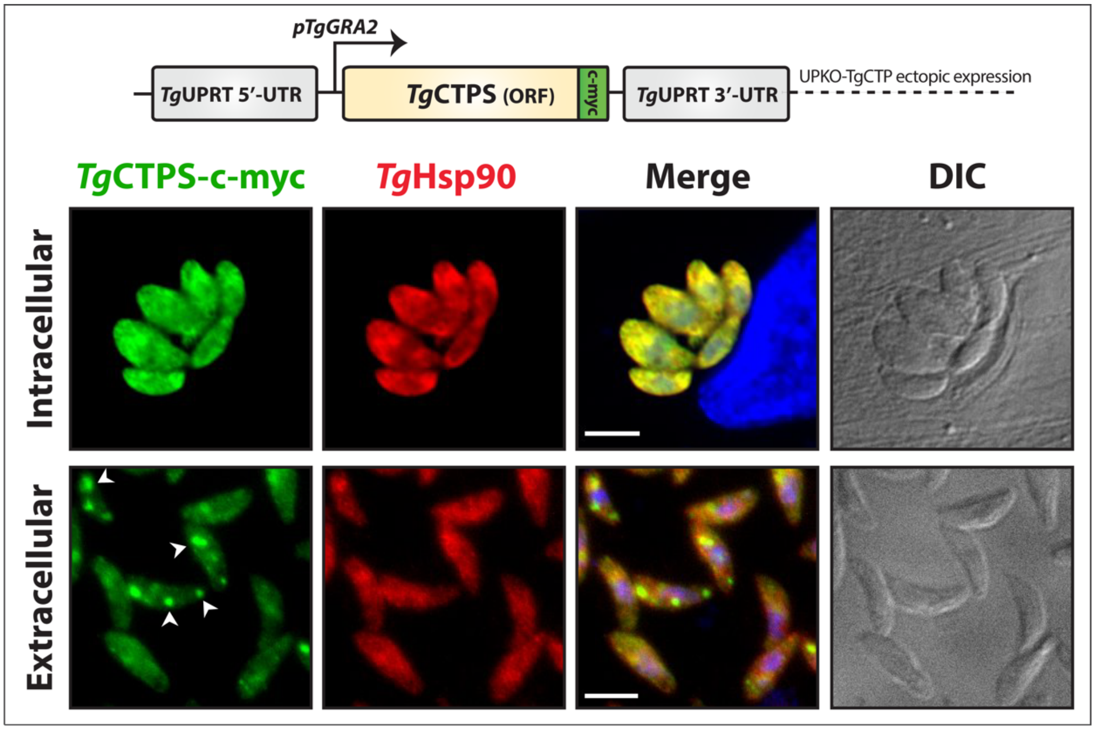

Supplementary Figure 5. **Ectopic expression of *Tg*CTPS in tachyzoites.** Ectopically expressed *Tg*CTPS under the *pGRA2* promoter. Immunostaining was performed using mouse anti-c-myc (green) and rabbit anti-TgHsp90 (red) antibodies. *Intracellular:* HFF cells were infected with transfected parasites and fixed 27 hpi. *Extracellular:* Fresh extracellular parasites were fixed using 4 % PFA. The *Tg*CTPS punctate distribution is indicated by arrowheads (white). DIC: differential interference contrast. Scale bars: 5 µm.

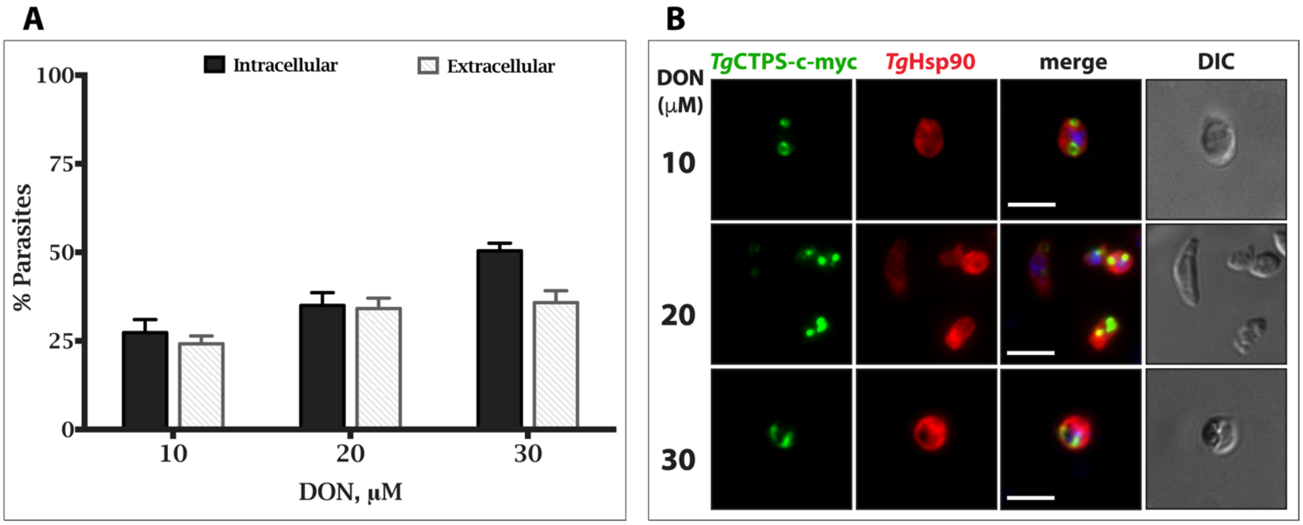

Supplementary Figure 6. Aberrant forms of intra- and extracellular parasites under DON treatment. **A.** The bars indicate the percent of parasites exhibiting an aberrant morphology with increasing amounts of DON, calculated based on the total number of cells for each condition. **B.** Spherical and shorter parasites were

observed, when fresh extracellular parasites were incubated 4 - 6 h in variable concentrations of DON. Scale bars: 5 µm.

A

E.C.	Enzyme	Gene ID	Pathway
6.3.5.2	GMP synthase (GMPS)	TGGT1_230450	Purine metabolism
6.3.5.5	Carbamoyl phosphate synthase (CPS II)	TGGT1_215260	Pyrimidine metabolism
6.3.5.4	Asparagine synthase	TGGT1_253430	Asparagine biosynthesis
6.3.5.1	NAD(+) synthase	TGGT1_269800	Nicotinamide metabolism
4.3.3.6	Pdx2 protein	TGGT1_281490	Pyridoxal biosynthesis
6.3.5.7	PET112 glutaminyl-tRNA synthase	TGGT1_233838	Aminoacyl-tRNA biosynthesis
2.6.1.16	Glucosamine fructuose-6-phosphate aminotransferase	TGGT1_231350	Hexosamine biosynthesis

B

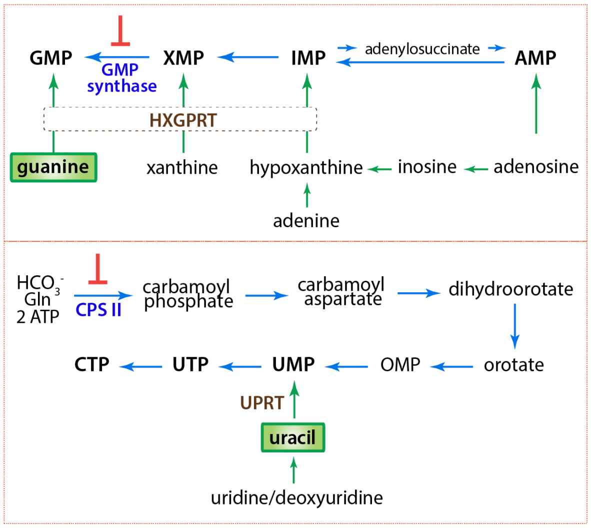

C

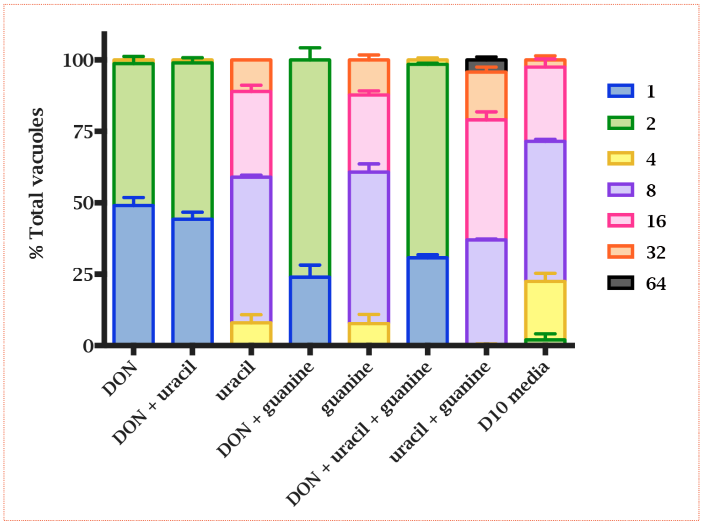

Supplementary Figure 7. Enzymes potentially affected by DON in *T. gondii*. (A) A table summarizing the glutaminases present in the *T. gondii* genome which could be inhibited when DON is added to the parasite culture. Three of them belong to nucleotide metabolism (6.3.4.2, CTP synthase; 6.3.5.2, GMP synthase and 6.3.5.5, carbamoyl phosphate synthase). Genes encoding for the putative proteins, PET112-glutaminyl tRNA synthase and glucosamine fructuose-6-phosphate amino transferase, are expressed at low levels in tachyzoites**. (B)** Scheme illustrating pathways to obtain GMP and UMP in *T. gondii*. Steps shown by blue arrows indicate reactions of interconversion or biosynthesis, whereas salvage pathway is represented by green arrows. Red lines indicate the enzyme inhibited by DON. Guanine was added to the culture to compensate for the inhibition of GMP synthase by DON (upper panel), and uracil was added to compensate for CPSII inhibition (lower panel). (**C)** Growth of parasites in media in the presence of 330 μM guanine, 200 μM uracil or 20

μM DON. The number of parasites per vacuole was counted 27 hpi in each evaluated condition. Data are represented as mean ± SD of three independent experiments.

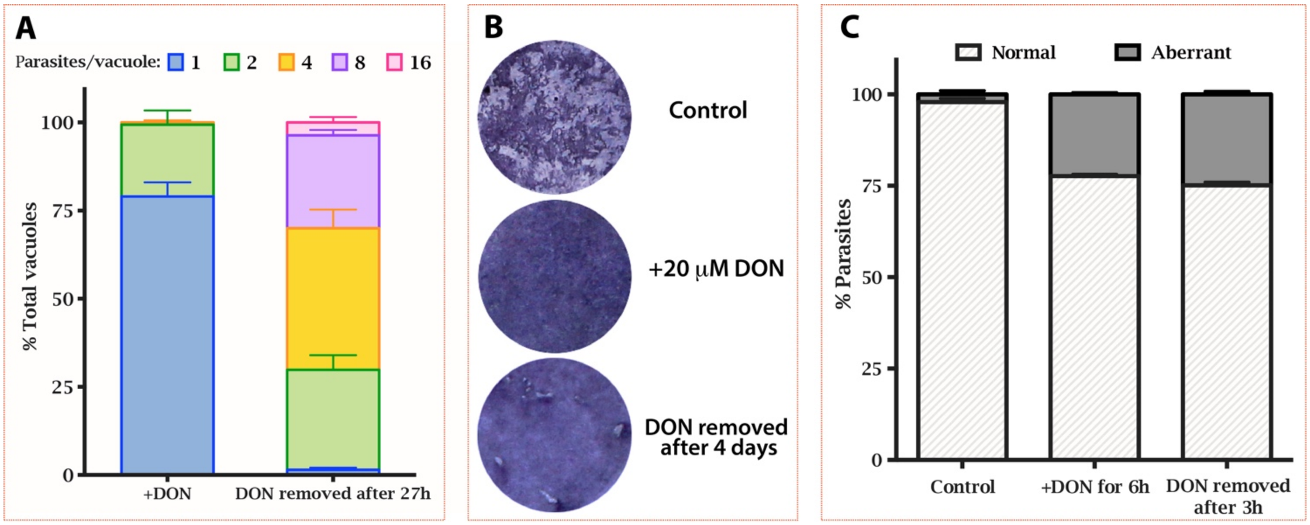

Supplementary Figure 8. DON inhibitor wash-out experiments on intra- and extracellular *T. gondii* tachyzoites. (A) Replication rate of parasites cultured in the presence of 20 μM DON for 27 h, followed by the removal of DON and incubation for another 27 h. **(B)** Plaque assay was performed in the absence of DON (top) and in the presence of 20 μM DON (middle). Plaques were fixed and stained after 8 days. For the wash-out experiment, infected cells were exposed to 20 μM DON for 4 days and then the inhibitor was removed by washing and replaced by D10 media for the next 4 days (bottom). **(C)** Quantification of the effect of DON on extracellular parasites. The bar graphs indicate the percent of parasites exhibiting a usual morphology compared to the percent of spherical and shorter parasites. All samples were scored after 6 h.

SUPPLEMENTAL TABLE

Primer name	Primer sequence (restriction site underlined)	Cloning vector
**Expression of *Tg*CTP in *E. coli***		
TgCTP_s<br>TgCTP_as	ATTACCATATGAACGCCGCCTCCCGAGCC<br>CCACATATGCTAGGCACTTTCCACCTCTTCGTAGACG	*pET-19b* (His-tag-*Tg*CTP in BL21 strain; 6xHis-*Tg*CTP)
TgCTP_truncated<br>TgCTP_as	CTCATCGTCGACCTAGGCACTTTCCACCTCTTC<br>CCACATATGCTAGGCACTTTCCACCTCTTCGTAGACG	*pET-15b* (His-tag-*Tg*CTP w/o 57aa of its N-terminal in BL21 strain)
**Subcellular localization of *Tg*CTP in *T. gondii***		
TgCTPS_F1<br>TgCTPS_R1	CTCATCATGCATAACGCCGCCTCCCGAGCC<br>CTCATCTTAATTAATTAcaggtcctcctcggagatgagcttctgctcGGCACTTTCCACCTCTTCGTAGACGGC	*pGRA2-UPKO* (For expressing an ectopic copy of *Tg*CTP-c-myc in tachyzoites)
TgCTP-IT-F<br>TgCTP-IT-myc-R	CTCATCCCACCGGTCACCTGGGCTCTCTCTCGATCTTTTCTGGT<br>CTCATCGAATTCcaggtcctcctcggagatgagcttctgctcGGCACTTTCCACCTCTTCG	*pTKO-HXGPRT* (For expressing TgCTP-c-myc under its own promoter in tachyzoites)
**Functional expression in *S. cerevisiae***		
TgCTP_F2<br>TgCTP_R2	CTCATCGCGGCCGCATGAACGCCGCCTCCCGA<br>CTCATCGCGGCCGCCTAGGCACTTTCCACCTCTTCGTAGA	*pESC-His or pNEV-N* (*TgCTP* in YBL039c or YJR103w strain)
ScCTP1_F<br>ScCTP1_R	CTCATCGCGGCCGCATGAAGTACGTTGTTGTTTCAGGT<br>CTCATCGCGGCCGCTTAAAAGTTGAATTTGTTTT	*pESC-His or pNEV-N* (*Sc*CTP1 in YBL039c strain)

	CGCC	
ScCTP2_F<br><br>ScCTP2_R	CTCATCGCGGCCGCATGAAATACGTTGTTGTTTC TGGT<br><br>CTCATCGCGGCCGCTCATTCATTTTCATTTCCCT CG	*pESC-His or pNEV-N* (*Sc*CTP2 in YJR103w strain)
TgCTP_His_F<br><br>TgCTP_R2	CTCATCGCGGCCGCATGGGCCATCATCATCATC ATC<br><br>CTCATCGCGGCCGCCTAGGCACTTTCCACCTCTT CGTAGA	*pNEV-N* (*TgCTP* in YJR103w strain)
TgCTP_F2<br><br>TgCTP_FLAG-R	CTCATCGCGGCCGCATGAACGCCGCCTCCCGA<br><br>CTCATCGCGGCCGCGGCACTTTCCACCTCTTCGT AGACG	*pESC-His* (Expression of *Tg*CTP-flag in YJR103w strain)
**Knockout of *Tg*CTP in *T. gondii* by Cre system**		
TgCTP_5'-UTR_Cre_F1<br><br>TgCTP_5'-UTR_Cre_R1	CTCATCGGGCCCCAAAACCGACCGAATTGATT<br><br>CTCATCGGGCCCCCAACAACGTGTGGGCAG	*pG140* (Complete knockout of *Tg*CTP by Cre recombinase in the *Δku80::diCre* strain)
TgCTP_3'-UTR_Cre_F1<br><br>TgCTP_3'-UTR_Cre_R1<br><br>TgCTP_3'-UTR_Cre_R2<br><br>TgCTP_3'-UTR_Cre_R3<br><br>TgCTP_3'-UTR_Cre_R4	CTCATCGAGCTCCATCCTCCATTTGCCTCTTT<br><br>CTCATCGAGCTCGTATTTGCGTTTGATGAAAGA ATACTT<br><br>CTCATCGAGCTCTGCAGCTGGCCAAAGTCC<br><br>CTCATCGAGCTCTCTCTCATCGCTTGAGTTCTCG<br><br>CTCATCGAGCTCTCTCTCATCGCTTGAGTTCTCG	*pG140* (Complete knockout of *Tg*CTP by Cre recombinase in the *Δku80::diCre* strain)

TgCTP_3'-UTR_Cre_B_R	CTCATCGAGCTCGAGTTTATCGACGACGAAGATAACT	
TgCTP_ORF_Cre_F1<br>TgCTP_ORF_Cre_R1	CTCATCCAATTG*CGACAAA*ATGAACGCCGCCTCCCGA<br>CTCATCTTAATTAATTAcaggtcctcctcggagatgagcttctgctcGGCACTTTCCACCTCTTCGTAGACG	*pG140* (Complete knockout of *Tg*CTP by Cre recombinase in the *Δku80::diCre* strain)
TgCTP_5Scr_Cre_F1<br>YFP_5Scr_Cre_R1	TAACTCTCTGGTTAGTCGCTCTTGT<br>CAGATGAACTTCAGGGTCAGC	Screening of 5'-crossover in transgenic *Δku80::diCre* strain
HX_3Scr_Cre_F1<br>TgCTP_3Scr_Cre_ R1<br>TgCTP-3Scr-Cre-R2<br>TgCTP-3Scr-Cre-R3<br>TgCTP-3Scr-Cre-R4<br>TgCTP-3Scr-Cre-B-R1<br>TgCTP-3Scr-Cre-B-R2	CTACGACTTCAACGAGATGTTCC<br>CATCAGAAACTCCTTTGGCC<br>GATGGCACAACTTGTTCGC<br>CATTTCTCGGTCTATATCTGTGCA<br>GAGTTTATCGACGACGAAGATAACT<br>GAAATTCGTTTGGAGTTGGTAGA<br>GTCTGTGTCTTTGTGGTTCAGC	Screening of 3'-crossover in transgenic *Δku80::diCre* strain
TgCTP_ISP_Cre_F1<br>TgCTP_ISP_Cre_R1	GTGTACGTCCTGGAGGACG<br>GCTCTGGGAGGACTTGAGTG	Comparison between g*Tg*CTP and c*Tg*CTP version in transgenic *Δku80::diCre* strain
**Knockdown of *Tg*CTP in *T. gondii* by 3'-UTR mRNA destabilization**		
TgCTP_LIC_Cre_F<br>TgCTP_LIC_Cre_R	TACTTCCAATCCAATTTAATGCTCTCTCTCGATCTTTTCTGG<br>TCCTCCACTTCCAATTTTAGCGGCACTTTCCACCTCTTCG	*pG152* (Knockdown of *Tg*CTP in the *Δku80::diCre* strain)

TgCTP_Scr_Cre 1 F<br><br>pG152/pG223-ESP-R1	CCATCTGCAAGGTGAGGAG<br><br>CTGTTATCCTGCAGTTACTTGTCG	*pG152* (Knockdown of *Tg*CTP in the *Δku80::diCre* strain)
**Conditional knockout of *Tg*CTP**		
TgCTP-ORF-myc-F1-UPKOtetsag<br><br>TgCTPS_R1	CTCATCTCATGAACGCCGCCTCCCGA<br><br>CTCATCTTAATTAATTAcaggtcctcctcggagatgagct tctgctcGGCACTTTCCACCTCTTCGTAGACGGC	*pNTP3TetO7Sag1* (Conditional knockout of *Tg*CTP in the *Δku80*-TaTi strain)
TgCTP_5'-UTR_Cre_F1<br><br>TgCTP-5UTR-pTet-CAT-R1	CTCATCGGGCCCCAAAACCGACCGAATTGATT<br><br>CTCATCGGGCCCCCAACAACGTGTGGGCAG	*pTub8CAT-GT1-KO* (Conditional knockout of *Tg*CTP in the *Δku80*-TaTi strain)
TgCTP-3UTR-pTet-CAT-F1<br><br>TgCTP-3UTR-pTet-CAT-R1	CTCATCCTCGAGGGCGCGTTCGAAAAAAGC<br><br>CTCATCTCTAGATCTCTCATCGCTTGAGTTCTCG	*pTub8CAT-GT1-KO* (Conditional knockout of *Tg*CTP in the *Δku80*-TaTi strain)
TgCTP_5Scr_Cre_F1<br><br>CAT-5Scr-R1	TAACTCTCTGGTTAGTCGCTCTTGT<br><br>TGCAGGAGAAAAAAATCACTGGA	Screening of 5'-crossover in transgenic the *Δku80*-TaTi strain
CAT-3Scr-pTet-F1<br><br>TgCTP-3Scr-Cre-B-R2	GTTTTCACCATGGGCAA<br><br>GTCTGTGTCTTTGTGGTTCAGC	Screening of 3'-crossover in transgenic the *Δku80*-TaTi strain
TgCTP-5UTR-pTet-DHFR-F1<br><br>TgCTP-5UTR-pTet-DHFR-R1	CTCATCCCACCGGTCACCTGGCAAAACC GACCGAATTGATT<br><br>CTCATCACTAGTCCAACAACGTGTGGGCAG	*pTKO-DHFR-TS* (Conditional knockout of *Tg*CTP in the *Δku80*-TaTi strain)
TgCTP-3UTR-pTet-DHFR-F1<br><br>TgCTP-3UTR-pTet-	CTCATCGTTAACGGCGCGTTCGAAAAAAGC<br><br>CTCATCGGGCCCTCTCTCATCGCTTGAGTTCTCG	*pTKO-DHFR-TS* (Conditional knockout of *Tg*CTP in the *Δku80*-TaTi strain)

DHFR-R1		
TgCTP_5Scr_Cre_F1<br><br>PSS2's 5-Scr-R	TAACTCTCTGGTTAGTCGCTCTTGT<br><br>CAAGGCGAGGTGAGACTGTG	Screening of 5'-crossover in transgenic the *Δku80*-TaTi strain
PSI/CDS-3Scr-F<br><br>PSSD2 –DHFR-F2<br><br>TgCTP-3Scr-Cre-B-R2	CTCGCTGGTAGTCCCAACTG<br><br>CTCGCGGCGTTGAATGTG<br><br>GTCTGTGTCTTTGTGGTTCAGC	Screening of 3'-crossover in transgenic the *Δku80*-TaTi strain

Table S1. Sequences of oligonucleotides used in this study. All sequences are presented 5' to 3'. Underlined sequence indicates a restriction site added to the primer. Sequence for the c-myc tag is shown in lower case. Lyophilized oligonucleotides were dissolved in ddH_2O (double-distilled water) at a concentration of 100 μM and stored at −20 ºC. Dilutions of 1:10 were made for working solutions.
